# Amyloid-β Precursor Protein APP Down-Regulation Alters Actin Cytoskeleton-Interacting Proteins in Endothelial Cells

**DOI:** 10.3390/cells9112506

**Published:** 2020-11-19

**Authors:** Emma Ristori, Vittoria Cicaloni, Laura Salvini, Laura Tinti, Cristina Tinti, Michael Simons, Federico Corti, Sandra Donnini, Marina Ziche

**Affiliations:** 1 Department of Life Science, University of Siena, 53100 Siena, Italy; emma.ristori@student.unisi.it; 2 Toscana Life Sciences Foundation, 53100 Siena, Italy; v.cicaloni@toscanalifesciences.org (V.C.); l.salvini@toscanalifesciences.org (L.S.); l.tinti@toscanalifesciences.org (L.T.); c.tinti@toscanalifesciences.org (C.T.); 3 Yale Cardiovascular Research Center, 300 George Street, New Haven, CT 06511, USA; michael.simons@yale.edu (M.S.); federico.corti@yale.edu (F.C.); 4 Departments of Medicine (Cardiology) and Cell Biology, Yale School of Medicine, New Haven, CT 06511, USA; 5 Department of Medicine, Surgery and Neurosciences, University of Siena, 53100 Siena, Italy

**Keywords:** amyloid-β precursor protein, APP, vascular APP, endothelial homeostasis, actin cytoskeleton-interacting proteins, integrins, Src/FAK, VEGFR2/VEGF

## Abstract

The amyloid-β precursor protein (APP) is a ubiquitous membrane protein often associated with Alzheimer’s disease (AD) and cerebral amyloid angiopathy (CAA). Despite its role in the development of the pathogenesis, APP exerts several physiological roles that have been mainly investigated in neuronal tissue. To date, the role of APP in vasculature and endothelial cells has not been fully elucidated. In this study, we used molecular and proteomic approaches to identify and investigate major cellular targets of APP down-regulation in endothelial cells. We found that APP is necessary for endothelial cells proliferation, migration and adhesion. The loss of APP alters focal adhesion stability and cell–cell junctions’ expression. Moreover, APP is necessary to mediate endothelial response to the VEGF-A growth factor. Finally, we document that APP propagates exogenous stimuli and mediates cellular response in endothelial cells by modulating the Scr/FAK signaling pathway. Thus, the intact expression and processing of APP is required for normal endothelial function. The identification of molecular mechanisms responsible for vasoprotective properties of endothelial APP may have an impact on clinical efforts to preserve and protect healthy vasculature in patients at risk of the development of cerebrovascular disease and dementia including AD and CAA.

## 1. Introduction

The amyloid-β precursor protein (APP) is a ubiquitous type-1 integral membrane protein that plays an important role in neurovascular degeneration [1]. APP is proteolytically cleaved by secretases, resulting in a series of biologically active fragments, including the amyloid-β peptide characterizing the pathogenesis of Alzheimer’s disease (AD) and cerebral amyloid angiopathy (CAA). Despite its major role in AD and CAA, APP cleavage products play diverse physiological roles that are important for neuronal development and function [2]. APP is involved in tissue repair after traumatic brain injury (TBI), exerting neuroprotective functions [3,4,5]. Furthermore, APP expression and processing are strongly increased in response to ischemic injury and stress exposure [6,7], suggesting a role in mediating tissue response to external perturbations.

At a cellular level, APP acts as a mechanical linker between the extracellular environment and the cellular cytoskeleton. Full-length APP functions as a membrane cell adhesion molecule in the developing nervous system by binding to several extracellular matrix components (HSPGs, reelin, laminin and F-spondin) and interacting with numerous adaptor proteins and other cell adhesion molecules (integrins, neural cell adhesion molecules) [8,9,10,11,12,13]. APP presents a conserved YENPTY cytoplasmatic domain similar to non-receptor tyrosine kinases; this motif interacts with scaffold proteins associated with the dynamics of the cytoskeleton and has been shown to regulate gene transcription. These features indicate that APP is involved in propagating extracellular responses [14]. Since neuronal damage is the main cause for cognitive decline in AD and CAA, much effort has been directed to understanding the role of APP in neuronal tissue; however, there is scarce information about its physiological role in other cell types including endothelial cells (ECs).

APP is highly expressed in the endothelium during embryogenesis, suggesting an important role for this macromolecule and its metabolites in vascular development and angiogenesis [15]. Genetic studies in zebrafish and mice revealed defects in vascular development and increased vulnerability to hypoxic injury upon APP gene inhibition [16,17]. More recently, APP was found to have protective properties in the vasculature by regulating expression of endothelial nitric oxide synthase (eNOS) in cerebrovascular endothelium [18].

Increasing evidence supports a hypothesis that vascular dysfunction precedes neuronal degeneration in AD and CAA development and that blood vessels are the origin for a variety of pathogenic pathways that lead to neuronal damage and dementia [19]. Thus, the understanding of the role of APP in ECs and in the vascular system homeostasis is critical to preservation of brain integrity.

Here, we investigated the biological role of APP in ECs homeostasis by generating an in vitro APP-knockdown model using human umbilical vein ECs (HUVECs). The loss of APP resulted in altered cellular morphology and reduction of cell migration and proliferation. We used a proteomic approach to identify biological pathways affected by APP silencing. This demonstrated altered protein expression of actin cytoskeleton-interacting proteins, mainly involved in actin organization, cell adhesion, cell–cell contact and VEGF-mediated angiogenesis in APP-deficient ECs. Integrins and other focal adhesion proteins also showed a reduced expression in ECs with reduced APP levels. Moreover, APP-silenced ECs lost barrier function due to altered expression of tight junctions proteins. Finally, we observed a reduced response to pro-angiogenic stimuli, due to a reduced activation of the VEGF signaling. Taken together, these results suggest that APP can regulate ECs responsiveness to extracellular environment by modulating cytoskeleton-interacting proteins.

## 2. Materials and Methods

### 2.1. Cell Cultures

Human umbilical vein endothelial cells (HUVECs) (Lonza, Basel, Switzerland) were used up to passage 7. Cells were cultured on 1% gelatin-coated dishes with endothelial growth medium (EGM-2) (Lonza) supplemented with antibiotics (100 U/mL penicillin and 100 μg/mL streptomycin, Euroclone, Milan, Italy), glutamine (2 mM, Euroclone), and 10% fetal bovine serum (FBS, Hyclone, GE Healthcare, Little Chalfont, UK).

### 2.2. Small Interfering RNA Transfection

Transient knockdown experiments were performed following the Reverse-Transfection of adherence cells protocol adapted from Qiagen (Qiagen, Hilden, Germany). Briefly, siRNAs and Lipofectamine^®^ 2000 (Invitrogen, Carlsbad, CA, USA) were diluted in Opti-MEM (Gibco, Thermo Fisher Scientific, Waltham, MA, USA) and incubated for 20 min at room temperature to allow the formation of the transfection complex. Confluent cells were then harvested and seeded on top of silencing complexes in 60 mm (3.5 × 10^5^ cells) or 100 mm (6.5 × 10^5^ cells) or 6-well plate dishs (1.8 × 10^5^ cells). Cells were transfected for 48 h under their normal growth conditions (EGM-2, 10% FBS) with 20 nM siRNA control (Negative Control siRNA, Qiagen #0001027310), or 20 nM siAPP-A (Hs_APP_8 FlexiTube^®^ siRNA, Qiagen #SI02776893), or 10 nM siAPP-B (Hs_APP_10 FlexiTube^®^ siRNA, Qiagen #SI02780288), or 20 nM siAPP-C (ON-TARGETplus Human APP (351) siRNA, Dharmacon #J-003731-06-0002). All results showed in main figures refer to HUVEC cells transfected with 20 nM of siAPP-C (Dharmacon Inc., Lafayette, CO, USA).

### 2.3. Real-Time PCR

The RNeasy Plus Kit (Qiagen) was used according to the manufacturer’s instructions to extract and prepare total RNA. A total amount of 1 μg RNA was transcribed, and quantitative RT-PCR was performed as previously reported [20]. The following primer sequences were used: APP forward 5′-TGGCCAACATGATTAGTGAACC-3′; APP reverse 5′-AAGATGGCATGAGAGCATCGT-3′; APLP1 forward 5′-CACCAGGTTGTGCCCTTCC-3′; APLP1 reverse 5′-GGCCTCACTCACAAATTCACC-3′; APLP2 forward 5′-CGACGGCACCATGTCAGAC-3′; APLP2 reverse 5′-CAACGAGGCATCACGGC-3′; ZO-1 forward 5′-GTGCCTAAAGCTATTCCTGTGAGTC-3′; ZO-1 reverse 5′-CTATGGAACTCAGCACGCCC-3′; claudin5 forward 5′-CTGCTGGTTCGCCAACATT-3′; claudin5 reverse 5′-TGCGACACGGGCACAG-3′; β-catenin forward 5′-GTCGAGGACGGTCGGACT-3′; β-catenin reverse 5′-CAAATCAGCTTGAGTAGCCATTGTC-3′; VE-cadherin forward 5′-GCACCAGTTTGGCCAATATA-3′; VE-cadherin reverse 5′-GGGTTTTTGCATAATAAGCAGG-3′; FAK forward 5′-ATCCCACACATCTTGCTGACTT-3′; FAK reverse 5′-GCATTCCTTTTCTGTCCTTGTC-3′; SRC forward 5′-CAGTGTCTGACTTCGACAACGC-3′; SRC reverse 5′-CCATCGGCGTGTTTGGAGTA-3′; VEGFR1, VEGFR2, VEGFR3 and ERK 1/2 (Qiagen); GAPDH forward 5′-GCCACATCGCTCAGACACC-3′; GAPDH reverse 5′-AATCCGTTGACTCCGACCTTC-3′. The fold change expression was determined using the comparative Ct method (2^−ΔΔCt^) normalized to GAPDH housekeeping gene expression. Data are reported as fold change relative to siCtrl, which was set to 1.

### 2.4. Western Blot

Cells were silenced for 48 h, harvested and seeded (3 × 10^5^ cells) in 6-cm plates. Media were then replaced with EGM-2; 10% FBS and lysate was collected after 24 h. For experiments with VEGF, cells were starved overnight in EBM-2, 0.1% FBS. After 24 h (60% of confluence), cells were treated or not with VEGFa (50 ng/mL, R&D Systems, Minneapolis, MN. USA #293-VE-025) for 5 min. Next, cells were washed and lysed, and an equal amount of proteins was used for Western analysis, as described [21]. The expression of APP (Cell Signaling, Danvers, MA, USA #2450, 1:1000), vinculin (Cell signaling #13901, 1:1000), paxillin (Millipore, Burlington, MA, USA #3794, 1:1000 and Abcam, Cambridge, United Kingdom #ab32084, 1:1000), Integrin β3 (Bioss, Woburn, MA, USA #bs0342R Rabbit, 1:1000), Integrin β1 (Santa Cruz, Dallas, TX, USA #SC6622, 1:1000), kindlin-3 (Cell Signaling #10459, 1:1000), ZO-1 (Thermo Fisher Scientific # 33-9100, 1:1000 and Life Technologies, Carlsbad, CA, USA #61-7300, 1:1000), VE-cadherin (Cell signaling #2500, 1:1000), β-catenin (Cell signaling #9562, 1:1000), claudin5 (Abcam, Cambridge, United Kingdom #ab53765, 1:300 and Thermo Fisher Scientific #35-250, 1:1000), p-VEGFR2 (Y1175, Cell Signaling #2478, 1:1000; Y951, Cell Signaling #4991, 1:1000; Y1059, Cell Signaling #3817, 1:1000), VEGFR2 (Cell Signaling #9698, 1:1000), p-ERK1/2 (T202/Y204, Cell Signaling #4370, 1:2000), ERK1/2 (Cell Signaling #9102, 1:1000), p-Src (Y416, Cell Signaling #6943, 1:1000), Src (Cell Signaling #2110, 1:1000), p-FAK (Y397, Sigma-Aldrich, St. Louis, MO, USA #8556, 1:1000), FAK (Cell Signaling #3285, 1:1000), β-actin (Sigma-Aldrich #A5316, 1:10000) and GAPDH (Cell Signaling #2118, 1:2000) were evaluated. Data are reported as fold change of arbitrary densitometry units (A.D.U.) of the target protein with respect to β-actin or GAPDH used as the loading control and normalized for siCtrl sample.

### 2.5. Immunoprecipitation (IP)

HUVEC cells were cultured in normal growth conditions until they reached confluence. Cells were quickly washed twice with ice-cold PBS, lysed in in 1% Triton lysis buffer and spun at 16,000× *g* for 20 min. 300 μg of cleared lysate was immunoprecipitated for 2 h at 4 °C under gentle rotation with 50 μL/sample of Protein-G DynaBeads (Thermo Fisher Scientific, Waltham, MA, USA), preincubated in 4 μg/sample of anti-VEGFR2 antibody (Cell Signaling #9698). Beads were washed 3 times with PBS, resuspended in 20 μL of 1× loading buffer and boiled for 10 min at 70 °C. Samples were analyzed by western blot as described above using anti-APP and anti-VEGF antibodies.

### 2.6. Immunofluorescence Microscopy

Cells were transfected with siRNA for 48 h as described above. Silenced HUVEC cells were then harvested and seeded (8 × 10^4^ cells) on 10 mm ø on glass coverslips pre-coated with 1% gelatin in triplicate in EBM-2 10% FBS. After 24 h cells were fixed with fresh 4% PFA for 10 min, blocked with 3% BSA for 40 min and incubated at 4 °C overnight in primary antibody. The following primary antibodies were used: rabbit anti-APP (Cell Signaling #2452, 1:100), mouse anti-APP (Cell Signaling #2450, 1:100); rabbit anti-vinculin (Cell signaling #13901, 1:100), rabbit anti-ZO-1 (Life Technologies #61-7300, 1:50), mouse anti-VE-cadherin (Santa Cruz #sc-9989, 1:200), mouse anti-claudin5 (Thermo Fisher #35-2500, 1:200), mouse anti- β-catenin (Cell Signaling #2677 1:200). The day after, cells were washed 3 times, 5 min each with 0.5% BSA in PBS and incubated for one hour at room temperature in secondary antibody: Alexa Fluor 488-labeled anti-Mouse (Thermo Fisher #A-11001, 1:400) and anti-Rabbit (Thermo Fisher #A-11008, 1:400) or Alexa Fluor 555-labeled anti-Mouse (Thermo Fisher #A32727, 1:400) and anti-Rabbit (Thermo Fisher #A32732, 1:400). For actin cytoskeleton staining, cells were incubated with conjugated DyLight 488-Phalloidin (Thermo Fisher #12379, 1:50) for 30 min at room temperature. DAPI (Thermo Fisher Scientific #62248, 1:1000) was used to counterstain nuclei. Stained cells were mounted and viewed by confocal microscopy (Leica SP5 with 63× oil objective, Leica, Wetzlar, Germany).

### 2.7. Cell Proliferation Assay

Cell viability was determined by MTT test [22] HUVECs were first silenced for 48 h. Transfected cells were then harvested and seeded in a 96-mutiwell plate (3 × 10^3^ cells/well) and incubated in EGM-2, 1% FBS for 18 h and 24 h. Cells were exposed for 4 h to 1.2 mM MTT (3-[4,5-dimethylthiazol-2-yl]-2,5-diphenyltetrazolium bromide; Sigma-Aldrich, St. Louis, MO, USA) in fresh PBS (without phenol red). After the solubilization of formazan crystals in DMSO, absorbance was measured with a microplate absorbance reader (Infinite 200 Pro SpectraFluor; Tecan, Männedorf, Switzerland) at 540 nm. Data are reported as the fold change of absorbance units (at 540 nm), taking as reference the siCtrl sample.

### 2.8. Wound Healing Scratch Assay

ECs migration was assessed using an in vitro wound healing assay as previously reported [20]. Briefly, cells were silenced for 48 h as previously described. Transfected cells were then harvested and seeded on a 24-mutiwell plate (1 × 10^5^ cells/well) and incubated under their normal growth conditions (EGM-2, 10% FBS) until they reached complete confluence (18–24 h). A sterile 1000 μL micropipette tip was used to scrape the confluent monolayer and create the wound. Wells were washed twice with PBS and cells were exposed to EBM-2 and the indicated treatment (0.1% FBS; 10% FBS or 50 ng/mL VEGFa). 2.5 mg/mL ARA-C (Cytosine β-D-arabinofuranoside; Sigma-Aldrich) was added to the wells to suppress cell proliferation. Images of the wound in each well were acquired from 0 h to 8 h, 18 h and 24 h under a phase contrast microscope at 10x magnification. Finally, cells were fixed and stained with the PanReac kit. Results were quantified using ImageJ software, and data are reported as % of scratch closure normalized to siCtrl.

### 2.9. Cell Adhesion Assay

HUVEC were silenced as previously described; after 48 h, cells were harvested and seeded in triplicate in 96-well plates (2 × 10^4^ cells/well) pre-coated with fibronectin (3 μg/mL) or collagen I (1 μg/mL) as previously described [23]. For short-term adhesion assays, attached cells were quantified after 1 h; for de-adhesion assays, cells were incubated for 18 h in normal growth conditions. At the end of the assay period, cultures were rinsed with two gentle washes with PBS (with Ca/Mg), fixed for 5 min with 4% paraformaldehyde and stained for 20 min with 0.5% Crystal Violet in distilled water for 20 min. Excess crystal violet was removed, and the cells were washed with water. The plates were dried and the stain extracted by 100 μL of methanol for 5 min. Absorbance was read at 540 nm in a plate reader (Infinite 200 Pro, SpectraFluor, Tecan, Männedorf, Switzerland).

### 2.10. In Vitro Permeability Assay

Cells were silenced for 48 h, harvested and seeded (2 × 10^5^ cells/well) on gelatin-coated insert membranes (0.4 μm diameter pores, Corning, New York, USA), and the inserts were placed in 12-multiwell plates and incubated for 48 h in normal growth conditions (EGM-2, 10% FBS). Confluent monolayers were then starved with EBM-2 with 0.1% FBS for 4 h and treated with IL-1β (10 ng/mL) for 6 h [24]. FITC-Dextran (3 kDa, 10 μm) was used as a fluorescent marker of permeability, which was evaluated after 15 min by measuring the fluorescence in a plate reader (Infinite 200 Pro, SpectraFluor, Tecan, Männedorf, Switzerland) at 485/535 nm (excitation/emission). Data are reported as fold change of fluorescence units (RFU), taking as reference the siCtrl sample in the condition of medium EBM-2 with the addition of 0.1% serum (control condition).

### 2.11. Tube Formation Assay

HUVEC were silenced for 48 h, harvested and seeded (3 × 10^4^ cells/well) on Corning^®^ Matrigel^®^-coated 48-well plate in EBM-2, 0.1% FBS medium (control condition) or VEGFa (50 ng/mL, R&D Systems #293-VE-025). After 8 h of incubation, ECs were photographed and network formation on Matrigel was measured by means of the number of mashes per field (Nikon Eclipse E400 and camera Nikon DS-5MC, Nikon, Melville, NY, USA).

### 2.12. LC-MS/MS Proteomic and Bioinformatic Analysis

Control and silenced HUVEC were lysed in 2% Sodium deoxycholate (SDC)/100 mM ammonium bicarbonate, reduced with 5 mM tris-(2-carboxyethyl)-phosphine (TCEP) and alkylated in the dark with 10 mM iodoacetamide (IAA). Protein quantification was assessed using Pierce-BCA protein assay kit. 60 µg of proteins, for each sample, were processed adding trypsin (1:40) and incubated at 37 °C overnight. All reaction mixtures were acidified with 1% formic acid (FA) [25,26]. Digested samples were desalted using OASIS cartridges and reconstituted in 0.1% formic acid in water/acetonitrile (97/3, *v*/*v*). LC-MS/MS analyses were performed using a Q-Exactive Plus Orbitrap mass spectrometer (Thermo Fisher Scientific). These experiments were carried out using a DDA setting to select the “top twelve” most-abundant ions for MS/MS analysis. MS data analysis was conducted using a Proteome Discover 2.1 (Thermo Fisher Scientific). Functional annotation analysis was performed using DAVID 6.8 [27] and FunRich 3.1.3 [28]. The default peak-picking settings were used to process the raw MS files in MaxQuant (version 1.6.1.0), Perseus (version 1.6.1.1) and its integrated search engine Andromeda [29,30]. The protein quantification and calculation of statistical significance was carried out using two-way Student-t test and error correction (*p* value < 0.05) with the method of Benjamini–Hochberg. For further visualization, a Principle component analysis (PCA) [31], a heatmap graphic analysis with clustering tree and, to detect differentially expressed proteins, a volcano plot analysis were performed.

### 2.13. Statistical Analysis

Results are expressed as means ±SEM. Statistical analysis was generated by GraphPad software Prism 7 (San Diego, CA, USA). Student’s *t*-test and Mann-Whitney U-test were applied to determined statistical differences between conditions. *p* < 0.05 was considered statistically significant.

## 3. Results

### 3.1. Loss of APP Affects Endothelial Cells Proliferation, Migration and Cytoskeleton Organization In Vitro

**Figure 1:**
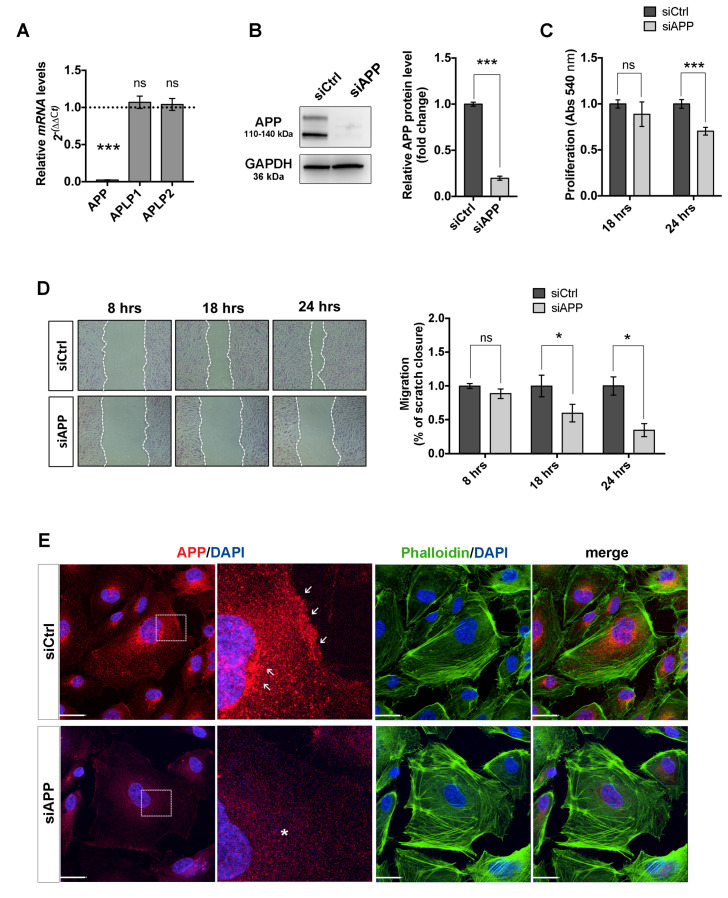
Amyloid precursor protein (APP) silencing reduces human umbilical vein EC (HUVEC) cell survival, migratory response and affects cytoskeleton organization: (**A**) mRNA expression levels are assessed by RTqPCR, relative mRNA levels of APP are significantly reduced upon silencing with siAPP for 48 h, while expression of homologues genes APLP1 and APLP2 is not affected, demonstrating the specificity of siRNA silencing with selected siAPP. GAPDH was used as housekeeping gene. Values are normalized to siCtrl (mean ± SEM; n = 3 replicates; *** *p* < 0.001, ns= not significant); (**B**) Western blot showing protein expression levels of APP following APP knockdown. Silencing of APP with siAPP-C for 48 h reduces APP protein levels. GAPDH was used as the internal control for western blotting. Data are presented as the means ± standard error of the mean (SEM); n = 5 replicates; *** *p* < 0.001; (**C**) MTT assay at 18 h and 24 h post transfection showing a reduction of cell proliferation of silenced cells compared to siCtrl starting from 24 h after transfection (mean ± SEM; n = 5 replicates; *** *p* < 0.001, ns= not significant); (**D**) Wound healing scratch assay at 8 h, 18 h and 24 h post transfection, showing a reduction of migration rate in HUVEC silenced for APP starting from 18 h after transfection (mean ± SEM, n = 5 replicates, * *p* < 0.05); (**E**) Immunofluorescence staining of control (siCtrl) and silenced (siAPP) HUVEC cells showing in red APP protein expression and in green F-actin stress fibers (Phalloidin-488), nuclei were stained with DAPI (blue). White arrows show APP high expression at the cell membrane and in the Golgi of siCtrl HUVEC; white asterisk show loss of APP expression in siAPP cells. Scale bar = 25 μm.

To study the physiological role of APP in ECs, we knocked down APP gene expression in HUVEC cells using an siRNA strategy. We started our experimental approach by selecting three different siRNAs, two targeting the 3’UTR region (siAPP-A and siAPP-B) and one targeting the APP mRNA coding sequence (siAPP-C). All three siRNAs (siAPP-A, siAPP-B and siAPP-C) knocked down significantly APP mRNA and protein expression after 48 h post transfection, as confirmed by RTqPCR analysis and western blot (WB) (Figure 1A,B and Figure S1A,B). In mammals, the APP gene family includes, besides APP, two genes encoding the APP-like proteins (APLP1 and APLP2) that share similar structural organization and partially overlapping functions [32]. To confirm the specificity of APP knockdown, we checked relative mRNA expression levels of APP homologues genes APLP1 and APLP2 upon silencing with siAPP-A, -B or -C respectively, and observed that siAPP-C was the only siRNA that didn’t show any off-target effect (Figure 1A and Figure S1C,D). Moreover, the siAPP-C was the only siRNA with no effect on cell survival, as both siAPP-A and siAPP-B significantly induced caspase-3 activation after 48 h transfection (Figure S1E,F). Taking into account these findings, we therefore used siAPP-C as the specific siRNA to knockdown APP (hereinafter referred to as siAPP).

We then investigated the effect of APP silencing on cell proliferation and migration of HUVEC cells. The MTT assay showed a reduction of cell proliferation 24 h after siRNA transfection in ECs silenced for APP (siAPP) compared to control siRNA treated cells (siCtrl) (Figure 1C). The wound healing scratch assay showed normal migration at 8 h after scratch and a significant reduction of migration rate at 18 and 24 h after scratch (Figure 1D).

To understand if the migratory defect was due to cytoskeletal disorganization, we labeled control cells (siCtrl) and APP-silenced cells (siAPP) with an anti-APP antibody and F-actin stress fibers marker (Phalloidin). APP immunoreactivity was observed at the cell membrane, in the cytoplasm and in the Golgi of siCtrl HUVEC, and was expectedly lost in siAPP cells. APP-silenced cells appeared bigger and flatter and the phalloidin staining showed an altered organization characterized by shorter and thicker actin stress fibers, suggesting a potential role of APP in controlling assembly, disassembly or rearrangement of cytoskeletal structures (Figure 1E).

Altogether, our data suggest that APP is involved in ECs migration, proliferation and a correct cytoskeleton organization.

### 3.2. Proteomic Analysis Confirms the Cytoskeleton Organization Defect and Reveals Alteration of Vascular Specific Pathways in APP-Silenced Cells

To investigate further the effect of APP on ECs biology, we performed a proteomic analysis to gain a comprehensive and quantitative description of changes in protein expression that occur in HUVEC cells upon APP silencing. We used a free-label quantification (LFQ) approach to directly compare relative abundance of proteins obtaining an unbiased insight into protein expression changes. To identify and quantify proteins, liquid chromatography followed by mass spectrometry (LC-MS) was performed on control HUVEC (siCtrl) and HUVEC silenced for APP (siAPP). Cells were silenced for APP for 48 h and samples were collected 24 h after siRNA transfection, maintaining the same experimental conditions of the results previously described.

Protein identification and quantification was performed with MaxQuant using the Uniprot_Homo sapiens (proteome:up000005640) database (search parameters were 20 ppm tolerance on peptides, 0.02 Da on fragments, and less than 1% false discovery rate, FDR). We identified 1225 and 1396 proteins for three biological replicates of siCtrl and siAPP, respectively (Figure S2A). To gain insight into the global similarities and differences between the six groups (siCtrl1, siCtrl2, siCtrl3 and siAPP1, siAPP2, siAPP3), we first performed an unsupervised clustering analysis that showed a differential clustering between siCtrl (siCtrl1, siCtrl2, siCtrl3) and siAPP (siAPP1, siAPP2, siAPP3) samples (Figure S2B). The principle component analysis (PCA) of all replicates of siCtrl and siAPP samples confirmed the clear proteomic differentiation between the two datasets (Figure S2C).

**Figure 2:**
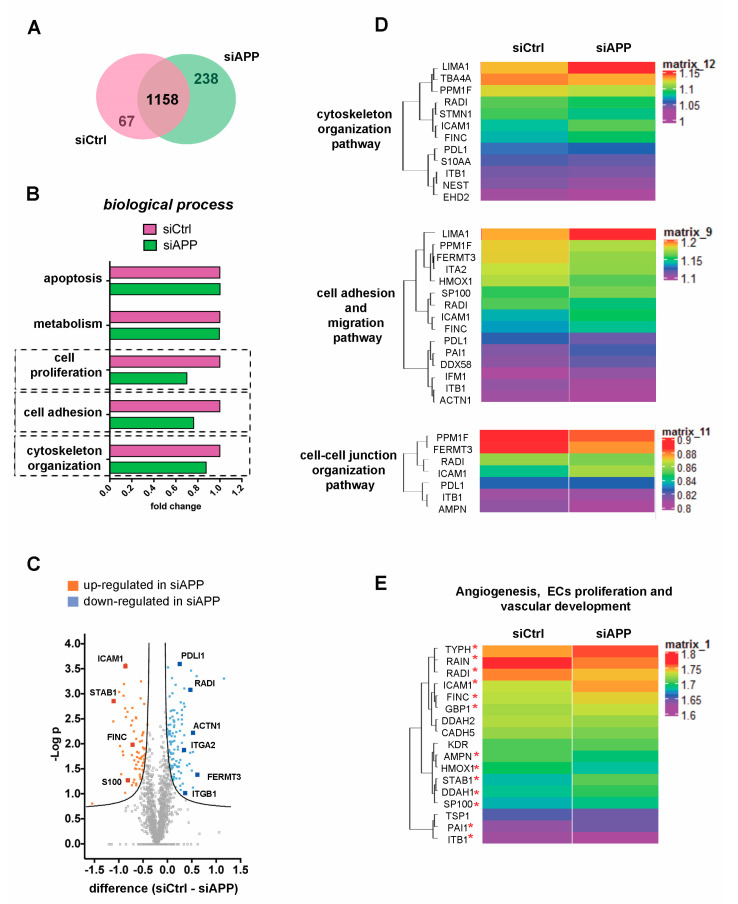
Proteomic analysis reveals a reduction of cytoskeleton and cell–cell, cell–extracellular matrix interaction proteins: (**A**) Venn diagram representing the number of common proteins in siCtrl and siAPP common datasets; (**B**) Functional comparison between siCtrl common dataset and siAPP common dataset using FunRich software reveals differences in cell proliferation, cell adhesion and cytoskeleton organization (*p*-value < 0.001). A fold change is calculated to show a functional comparison and reveals a reduction in the percentage of proteins involved in different biological processes; (**C**) The Volcano plot was constructed to show the significantly differentially expressed proteins between two datasets. Proteins with statistically significant differential expression (siCtrl–siAPP ≥ ±1.5, FDR < 0.05) are located in the top right and left quadrants. The orange points represent significantly upregulated proteins in siAPP and the blue points represent significantly downregulated proteins in siAPP; (**D**) The heat maps show proteins with statistically significant differential expression; (**E**) The heat map shows the differential expression of proteins present in siAPP and siCtrl common database and specifically involved in vascular development, angiogenesis and ECs proliferation. Only proteins with red asterisk show statistical significance.

We then performed a functional comparison between the siCtrl common dataset and siAPP common dataset using FunRich software to analyze differences (*p* value < 0.001) in cellular components and biological processes (Figure 2A,B and Figure S2D). We observed a reduction, in the expression of proteins involved in membrane (GO:0016020) and cytoskeleton (GO:0005856) cellular components (Figure S2D). In line with the previously described functional data, we observed a decrease of proteins associated with cell proliferation (GO:0008283) in the siAPP sample compared to the control (Figure 2B). We also found an evident reduction of proteins involved in cytoskeleton organization (GO:0030036) in the silenced sample (Figure 2B), confirming the defect in actin cytoskeleton of siAPP HUVEC cells previously described (Figure 1E).

The differential abundance of the 1158 common proteins was illustrated by volcano plot. A two-way Student-*t* test (*p* value < 0.05) was employed using Perseus software (v 1.6.1.1) to define the proteins that were differentially regulated in siAPP and siCtrl samples. The following criteria were applied: siCtrl–siAPP ≥ ±1.5, permutation-based FDR value set at 0.05. Proteins with a p-value less than 0.05 were considered statistically significant. We quantified 68 up-regulated and 105 down-regulated proteins in the siAPP HUVEC compared to the control group (siCtrl) (Figure 2C, Table S1). We identified a significant differential expression of proteins mainly involved in actin cytoskeleton organization (GO:0030036), in cell adhesion (GO:0007155) and in cell–cell junction organization (GO:0045216). In particular, we observed a down-regulation of actin-binding proteins involved in cytoskeleton regulation and dynamics such as radixin (RADI), α-actinin1 (ACTN1) and DPLI-1 and of proteins involved in promoting endothelial cell adhesion and migration such as integrins (ITGA2; ITGB1), and the integrin-activator kindlin3 (FERMT3) (Figure 2C,D).

We finally assessed differential expression of proteins involved in angiogenesis and vascular development and observed that endothelial markers such as VEGFR-2 (KDR) and VE-cadherin (CADH5) were not affected by the APP knockdown (Figure 2E). However, therefore was an up-regulation of endothelial activation markers such as ICAM-1, s-100 (S100), stabilin-1 (STAB1) and Fibronectin (FINC), indicating a role of APP in vascular inflammation (Figure 2E).

Collectively, these data confirm the presence of proliferation, migration and cytoskeleton organization defects observed in siAPP HUVEC and identify a significative change in ECs anchoring to the extracellular matrix, as well as in the interaction between adjacent ECs. This suggests the involvement of APP in vascular stability, integrity, and responsiveness to exogenous stimuli.

### 3.3. APP Silencing Affects Endothelial Cells Adhesion and Promotes a Reorganization of Focal Adhesions Complex

**Figure 3:**
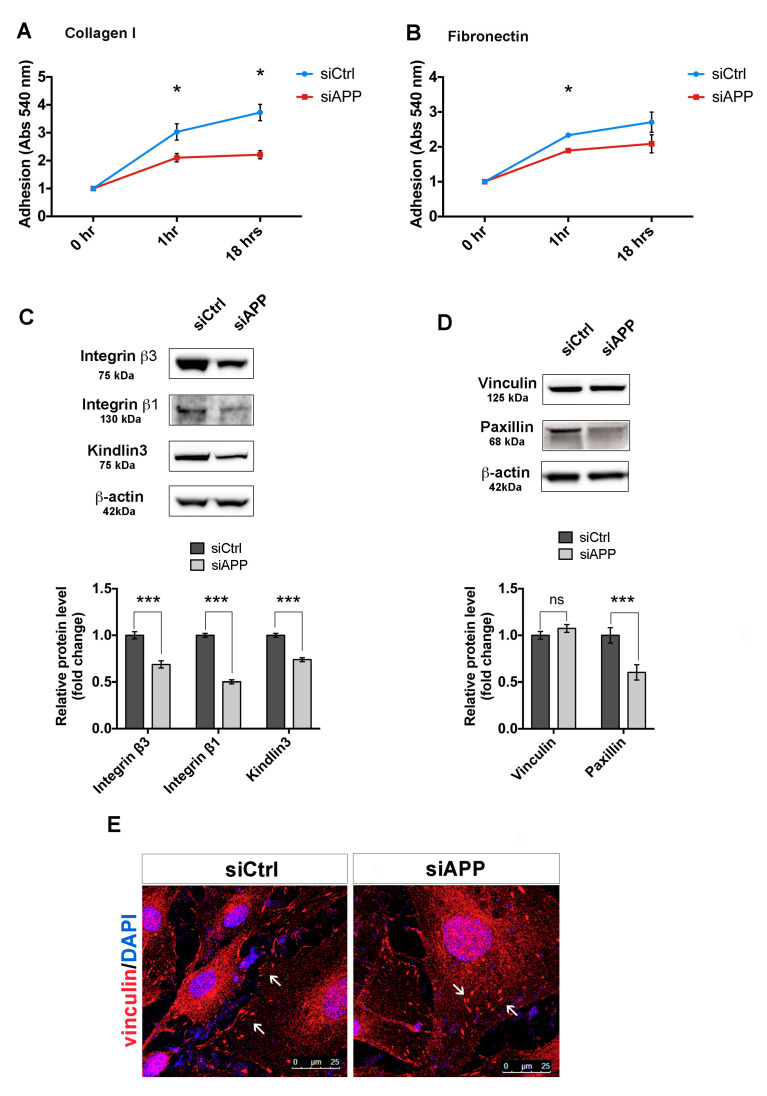
APP silencing affects HUVEC cell adhesion and focal adhesion organization and expression: Adhesion assay performed on collagen I (1 μg/mL) (**A**), and fibronectin (3 μg/mL) (**B**) coatings at different time-points (1 h, “short term adhesion”, and 18 h “de-adhesion”) (triplicate wells, n = 3). Attached cells were quantified by Crystal Violet staining and absorbance (Abs) measured at 540 nm wavelength. Values are represented as mean ± SEM; * *p* < 0.05; (**C**) Western blot analysis of integrin β1, integrin β3 and kindlin3 protein expression in control and silenced HUVEC cells. The bar graph shows quantification of 4 replicates (n = 4). β-actin was used as the internal control. Data are presented as the means ± SEM, *** *p* < 0.001; (**D**) Western blot analysis of focal adhesion proteins in control and silenced HUVEC cells. The bar graph represents the fold changes of the relative levels of focal adhesion proteins normalized to siCtrl. β-actin was used as the internal control. Data are presented as the means ± SEM, n = 4 replicates, *** *p* < 0.001, ns= not significant; (**E**) Subconfluent siCtrl and siAPP HUVEC cells stained for vinculin. White arrows indicate abnormal vinculin localization in siAPP cells compared to siCtrl. Scale bar = 25 µm.

To confirm our proteomics results, we further investigated the role of APP on HUVEC cell-matrix adhesion and focal adhesions organization. We first assessed siAPP cell-matrix adhesion by investigating cell attachment on two different integrin’s substrates, collagen I and fibronectin. HUVECs use predominantly α1β1 and α2β1 to adhere to collagen I and α5β1, α4β1 and αVβ3 to adhere to fibronectin [33]. Loss of APP prevented HUVEC cell attachment on both substrates (Figure 3A,B).

Moreover, loss of APP led to a reduction of protein expression of integrin β1, integrin β3 and the integrin activator kindlin-3 (FERMT3) in siAPP cells, validating our proteomic results (Figure 3C).

Integrin-dependent cell adhesion is predominantly mediated by focal adhesion proteins that link the integrins domains to the actin cytoskeleton to form the adhesion complex [34]. Thus, the down-regulation of integrin β expression might result in an altered organization of other focal adhesion components. To test this, we analyzed the expression levels of paxillin and vinculin, important components of focal adhesions [35]. Western blot analysis showed reduced protein expression levels of paxillin, but not vinculin in siAPP HUVEC (Figure 3D and Figure S4). However, while siCtrl cells showed a clear localization of vinculin to the cell membrane, siAPP cells presented a less organized expression (Figure 3E, arrows).

Taken together these results demonstrate that loss of APP interferes with the integrin β-mediated cell adhesion by modulating focal adhesion proteins expression and organization.

### 3.4. APP Controls Endothelial Barrier Function

Integrin-mediated cell-matrix adhesion, as well as endothelial cell junctions-mediated cell–cell adhesion, is essential for maintenance of endothelial monolayer barrier function [36,37,38].

**Figure 4:**
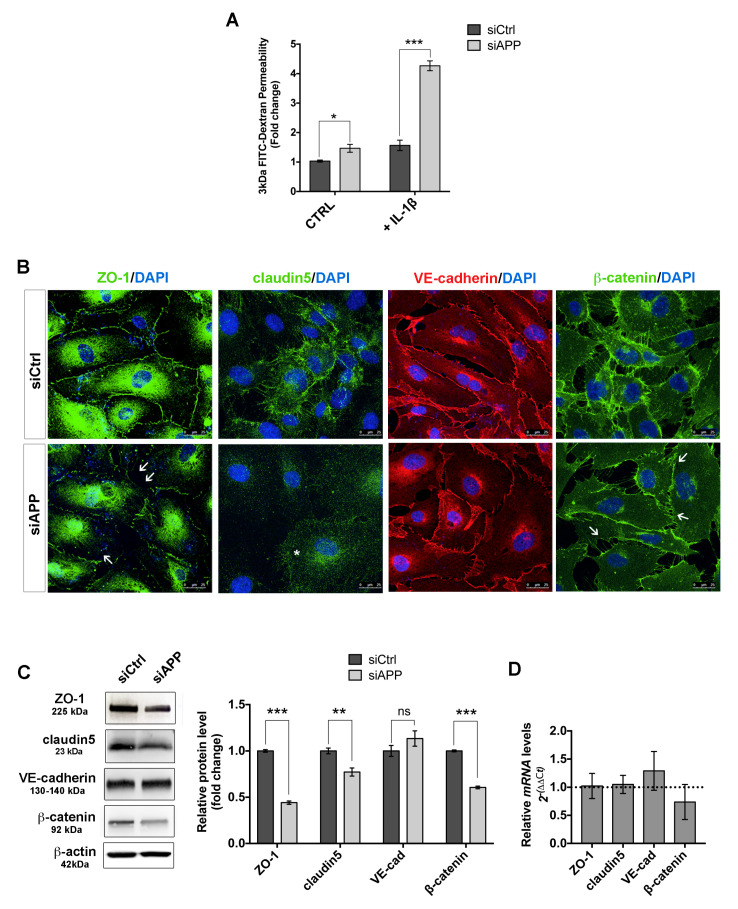
Loss of APP affects HUVEC monolayer barrier function: (**A**) Silenced and control HUVEC were grown on gelatin-coated insert membranes for 48 h after transfection to ensure confluence and treated with or without IL-1β (10 ng/mL) for 6 h. Permeability was measured using the tracer molecule FITC-dextran (3 kDa). Data are reported as fold change relative to siCtrl in basal conditions (mean ± SEM, n = 3 replicates, * *p* < 0.05, *** *p* < 0.001); (**B**) Confluent silenced and control HUVEC were labeled for ZO-1 (green), claudin5 (green), VE-cadherin (red) and β-catenin (green). Staining shows a decreased claudin5 expression (asterisk) and reduced ZO-1 and β-catenin expression and organization at the cell–cell contact sites (white arrows) in cells silenced for APP. Scale bar = 25 μm; (**C**) Western blot analysis of cell–cell junction proteins expression. The bar graph shows a significative reduction of ZO-1, claudin5 and β-catenin protein expression upon APP silencing (mean ± SEM, n = 3 replicates, ** *p* < 0.01, *** *p* < 0.001, ns= not significant); (**D**) RTqPCR showing unchanged mRNA expression.

Our proteomic data showed a reduced abundance of proteins involved in cell–cell junction organization, indicating a possible defect in permeability in HUVEC cells lacking APP. To test this, we performed an in vitro permeability assay using confluent monolayers of siCtrl and siAPP HUVECs. We observed a significant increase of cellular permeability in HUVECs lacking APP under basal conditions (CTRL). To stress the system, we treated cells with interleukin-1 beta (IL-1 β), a pleotropic cytokine known to induce endothelial permeability [39]. IL-1β (10 ng/mL, 6 h) induced a significant increase in paracellular flux of FITCH-dextran in both siCtrl and siAPP cells; however, siAPP HUVEC cells showed an almost 4 fold-change increase when compared to siCtrl (Figure 4A). These results suggest a higher susceptibility to pro-inflammatory stimuli of ECs in the absence of APP.

Endothelial cell junctions, such as adherens junctions and tight junctions, have a critical role in maintaining vascular integrity. Modifications in the expression and organization of these cellular components increase endothelial permeability and vascular fragility in vivo [40,41]. We, therefore, assessed the expression and organization of the tight junction proteins ZO-1 and claudin5 and of the adherens junction proteins VE-cadherin and β-catenin in siAPP confluent HUVEC.

Immunostaining for ZO-1 and claudin5 revealed a reduction in tight junctions and ZO-1 localization at cell–cell contacts of siAPP HUVEC (Figure 4B, asterisk and white arrows). We also observed a reduction in the intensity of β-catenin staining, but no obvious defect in VE-cadherin expression and distribution (Figure 4B, white arrows). The reduction in the total amount of ZO-1, claudin5 and β-catenin protein levels was further confirmed by western blot analysis (Figure 4C and Figure S4). mRNA expression levels were not affected by loss of APP, suggesting a defect in proteins stability (Figure 4D). Although VE-cadherin expression was unaffected by the silencing, the reduction of β-catenin protein levels indicated a defect in VE-cadherin activity. Altogether these data suggest that APP is involved in modulating the stability and localization of endothelial cell junctions.

### 3.5. APP Modulates Endothelial Response to Pro-Angiogenic Stimuli

Integrins and monolayer integrity are essential for vascular tissue homeostasis and endothelial responsiveness to angiogenic factors. We hypothesized that loss of APP, and the consequent deregulation of integrin expression would reduce endothelial cell ability to respond to extracellular stimuli including growth factors, thus negatively affecting angiogenesis.

**Figure 5:**
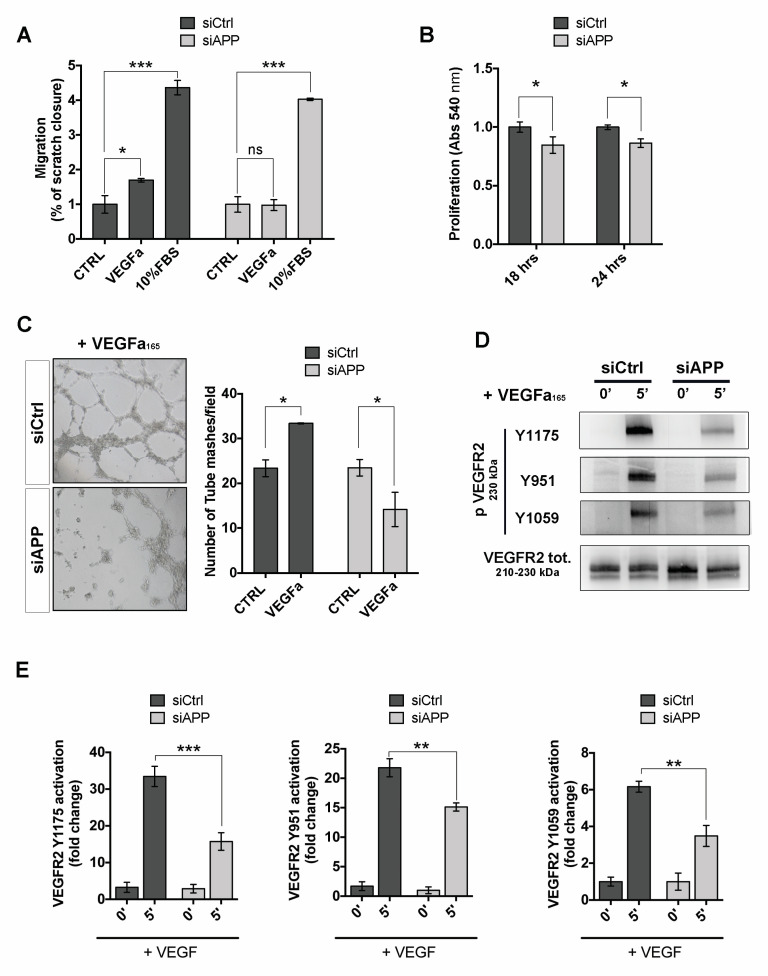
APP controls HUVEC response to angiogenic factors and modulates VEGFR2 activation: (**A**) Wound healing scratch assay at 8 h post transfection, showing a reduction of migration rate in HUVEC silenced for APP exposed to VEGFa (50 ng/mL) stimulation but not to 10% fetal bovine serum (FBS) (mean ± SEM, n = 4 replicates, * *p* < 0.05, *** *p* < 0.001, ns= not significant); (**B**) MTT assay at 18 h and 24 h post transfection of VEGFa treated cells (50 ng/mL, 18 and 24 h respectively) showing a reduction of cell proliferation of silenced cells with siAPP compared to siCtrl (mean ± SEM; n = 3 replicates; * *p* < 0.05); (**C**) Tube formation assay showing a reduced angiogenic response of siAPP cells. On the left, representative pictures of HUVEC network (10× magnification); On the right, quantification of number of mashes per picture (mashes/field) of silenced and control HUVEC seeded on Matrigel coating and treated with VEGFa (50 ng/mL) for 8 h. The results represent the mean ± SEM of 5 pictures (fields), * *p* < 0.05; (**D**) Western blot analysis showing evident reduction of VEGFR2 activation (phospho VEGFR2 Y1175, Y951 and Y1059) in APP-silenced cells stimulated with VEGFa (50 ng/mL) for 5 min; (**E**) Quantification of the immunoblots in c), The bar graphs represent the relative level of p-VEGFR2 Y1175, Y951 or Y1059 over total VEGFR2 in siCtrl and siAPP samples as determined by band density analysis (mean ± SEM, n = 4 replicates, ** *p* < 0.01, *** *p* < 0.001).

To investigate the effect of APP silencing on angiogenesis we stimulated siCtrl and siAPP HUVECs with VEGF and measured VEGF-mediated cell migration and cell proliferation. The wound healing scratch assay showed that APP silencing significantly inhibited cell migration in response to VEGF at 8 h after scratch. Interestingly, silenced cells migrated normally in presence of 10% FBS, indicating that the defect observed was due to a dysregulation of VEGF-mediated signaling (Figure 5A). Moreover, silencing of APP also reduced the cell proliferation in response to VEGF treatment (Figure 5B).

We then investigated the ability of siAPP HUVEC seeded on Matrigel layer to form capillary-like tube structures in response to VEGF (50 ng/mL, 8 h). VEGF significantly promoted tube formation in siCtrl sample, while the formation of web/net-like structures was significantly inhibited in siAPP cells (Figure 5C).

To further support the hypothesis that the loss of APP affects the VEGF-mediated signaling, we analyzed activation of the VEGF_receptor-2 (VEGFR2) in response to VEGF. We observed that APP silencing significantly suppressed VEGFR2 phosphorylation following VEGF stimulation at its major phosphorylation sites, located, respectively, in the kinase insert domain (Y951), in catalytic domain (Y1054/1059), as well as in the carboxy-terminal domain (Y1175). Interestingly, we did not observe a decrease of VEGFR2 total protein and mRNA expression (Figure S3A,B). Notably, VEGFR2 was unable to immunoprecipitate APP, suggesting that VEGFR2 and APP do not interact or form a complex (Figure S3C). Taken together, these results suggest that APP controls ECs response to VEGF by indirectly modulating VEGFR2 activation.

### 3.6. APP Modulates Src/FAK Signaling

**Figure 6:**
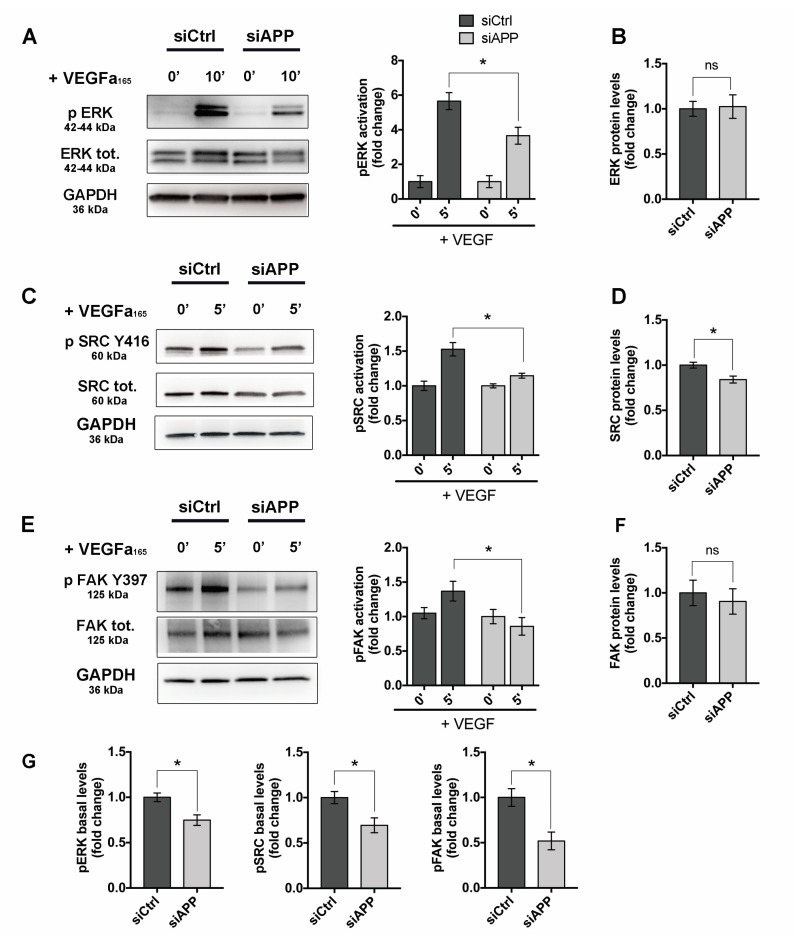
APP knockdown suppresses Src/FAK signaling: (**A**) Western blot analysis of ERK activation (p-ERK) shows reduced ERK phosphorylation in siAPP HUVEC treated with VEGF (50 ng/mL, 10 min) (**B**) Quantification showing unchanged ERK total levels upon APP silencing; (**C**) Western blot analysis of Src activation (p-Src) after VEGF treatment (50 ng/mL, 5 min) shows a significative reduction of p-Src-Y416 phosphorylation; (**D**) Quantification showing significative reduction of Src total levels in siAPP HUVEC; (**E**) Western blot analysis of FAK activation (p-FAK) after VEGF treatment (50 ng/mL, 5 min) showing down-regulation of FAK Y397 phosphorylation; (**F**) Quantification showing unchanged FAK total protein levels; (**G**) Quantification showing reduction of basal levels of p-ERK, p-SRC (Y416) and p-FAK (Y397) in siAPP HUVEC. All data are represented as mean ± SEM, n = 4 replicates; * *p* < 0.05, ns= not significant.

We next investigated the activation of VEGF–VEGFR2 downstream signaling in APP-silenced cells. HUVEC cells were transfected with siCtrl or siAPP for 48 h and then stimulated with VEGF (50 ng/mL). VEGFR2 phosphorylation at Y1175 site promotes ECs proliferation and migration through ERK pathway activation. Consistently with VEGFR2 Y1175 reduced activation, we found a significant reduction in p-ERK in siAPP HUVEC treated with VEGF, while total ERK protein and mRNA levels were unchanged (Figure 6A,B and Figure S3B).

VEGFR2 phosphorylation by VEGF also activates the Src/FAK pathway to promote ECs migration and adhesion. However, Src can be also activated by integrins binding to extracellular matrix, making the Src/FAK pathway a meeting point between growth factors-mediated and integrins-mediated ECs proliferation, migration and survival.

We found that phosphorylation of Src at Y416 site, as well as phosphorylation of FAK at one of the Src-target sites (Y396) were significantly reduced in APP-silenced HUVEC when exposed to VEGF (Figure 6C,E). Total Src protein levels, but not mRNA levels were reduced by APP knockdown (Figure 6D and Figure S3B). FAK total protein expression was unchanged, while mRNA levels were slightly up regulated by APP silencing, probably due to a compensatory mechanism (Figure 6F and Figure S3B).

Notably, phosphorylation of ERK, Src and FAK was significantly reduced also at basal conditions in siAPP HUVEC, suggesting that APP regulates focal adhesion components phosphorylation independently of VEGFR2 activation (Figure 6G).

Taken together these data strongly indicate that APP controls Src/FAK pathway.

## 4. Discussion

The amyloid precursor protein (APP) is a membrane bound protein present in multiple cell types, including ECs. However, its functional role in the vasculature is still unknown. Previous studies suggest that APP may exert a protective role by regulating eNOS expression in cerebral vasculature. Yet, the phenotype of ECs lacking this protein is poorly known [42]. In the present study, we show that APP has an important role in endothelial cell function by modulating expression of cytoskeleton-interacting proteins and mediating endothelial cell responses to extracellular stimulation. Indeed, the loss of APP in ECs resulted in a defective actin cytoskeleton organization with a consequent reduction of cell adhesion, migration, proliferation and barrier function, and in inhibition of response to the pro-angiogenic growth factor VEGF-A.

Proteomic results showed that cellular compartments most strongly affected by the loss of APP are the plasma membrane and cytoskeleton. In particular, proteins involved in cell adhesion (FERMT3; ITGA2; ITGB1) and actin cytoskeleton organization (RADI; DPLI-1; ACTN1) were downregulated, suggesting that APP acts in ECs to maintain cellular functionality. Furthermore, protein markers of endothelial activation and dysfunction were upregulated (ICAM-1, S100, STAB1 and FINC), indicating that APP is instrumental for physiological endothelial-dependent vascular homeostasis.

We further demonstrated that the loss of APP resulted in a reduced expression of integrin-β1 and integrin-β3. Moreover, both proteomic and molecular results showed a down-regulation of the integrin-activator kindlin3 (FERMT3), indicating that APP not only controls expression and/or the stability of integrins in ECs expression, but also their activation.

Integrins activity is essential for ECs homeostasis [33,43,44]. As part of the focal adhesion complex, integrins provide not only a mechanical linkage between the intracellular actin cytoskeleton and extracellular matrix, but also mediate bidirectional signaling “outside-in” and “inside-out” through the cell membrane. Kindlins participate in linking integrins to the actin cytoskeleton, mediating the “inside-out” signal from the cytoplasm to the extracellular integrin domain [45,46]. Interestingly, kindlins have been shown to modulate APP metabolism and a genome-wide study on Alzheimer’s related genes identified kindlins as a genetic risk factors [47].

Dysfunction of integrin-mediated signaling was confirmed by reduction of paxillin expression and loss of Src and FAK activation in siAPP cells. Paxillin and Src/FAK mediate the “outside-in” signal and their activation is critical for the stabilization of cell adhesion and the promotion of cell migration, proliferation and survival. In response to ligand-integrin binding, FAK auto-phosphorylates (Y397) and promotes Src activation that, in turn, phosphorylates FAK (Y576/577), promoting its kinase activity and its interaction with paxillin. Paxillin regulates gene expression through MAPK cascade and it is responsible to changes in shape and reorganization of the actin cytoskeleton by binding to downstream kinases and adaptors including the actin-binding protein vinculin [48]. Accordingly, in our study the reduction of paxillin expression coincides with an altered distribution of vinculin in siAPP cells.

Deregulation of the integrin-mediated signaling can also be responsible for the loss of endothelial barrier function. A recent study showed that the loss of integrin β1 and the consequent disruption of integrin β1-matrix interaction increase cerebral microvascular endothelial cell monolayer permeability in vivo and in vitro through reorganization of tight junction proteins via altered F-actin conformation [43]. Accordingly, in siAPP HUVECs we observed a decreased ZO-1 and claudin5 expression, associated with a reduction of integrin β1 expression and a consequent increase of endothelial permeability. Furthermore, in agreement with the notion that ZO-1 works as a major cytoskeleton organizer in ECs by controlling F-actin fiber distributions and regulating the tensile force acting on VE-cadherin [41], we also observed a decreased β catenin expression and altered VE-cadherin localization

Finally, we showed that the reduced expression of the focal adhesion complex and its altered interaction with actin-cytoskeleton, affects the ability of ECs to respond to the VEGF growth factor. We observed a reduced activation of VEGR2 receptor in response to exogenous VEGF in siAPP HUVEC and consequent inhibition of VEGF-induced endothelial cell migration and proliferation and loss of ECs angiogenic potential. We speculate that APP modulation of VEGF/VEGFR2 signaling is mediated by APP-integrin interaction since APP can interact with integrin-β1 [13,49], however further investigations are needed to elucidate this mechanism.

Here, we observed that siAPP HUVEC express lower levels of integrin-β3 and this coincides with lower levels of activation of the receptor. It is well known that integrin-β3 promotes VEGF/VEGFR2 signaling and that integrin KO models result in inhibition of angiogenesis and endothelial dysfunction [44,50,51]. However, the reduced response to VEGF might also be due to the alteration of shape and disorganization of the actin cytoskeleton in absence of APP. Indeed, the correct phosphorylation and stability at the cell membrane of the VEGF2 receptor is mediated by the correct interaction with cytoskeleton and cell surface proteins [52,53,54].

The downstream signaling of integrins and growth factors converge on the Src/FAK pathway. We showed that Src/FAK signaling was suppressed upon VEGF treatment in absence of APP. However, we also observed an inhibition of p-Src, and p-FAK in siAPP cells in basal condition, suggesting that the effect of APP on Src/FAK signaling is VEGF-independent.

Similarly, the loss of APP inhibits ERK1/2 activation independently from VEGFR2 activation. Indeed, APP can directly activate ERK1/2 signaling through its intracellular domain (AICD), and ERK1/2 phosphorylation/activation is increased in AD [55,56,57,58].

**Figure 7:**
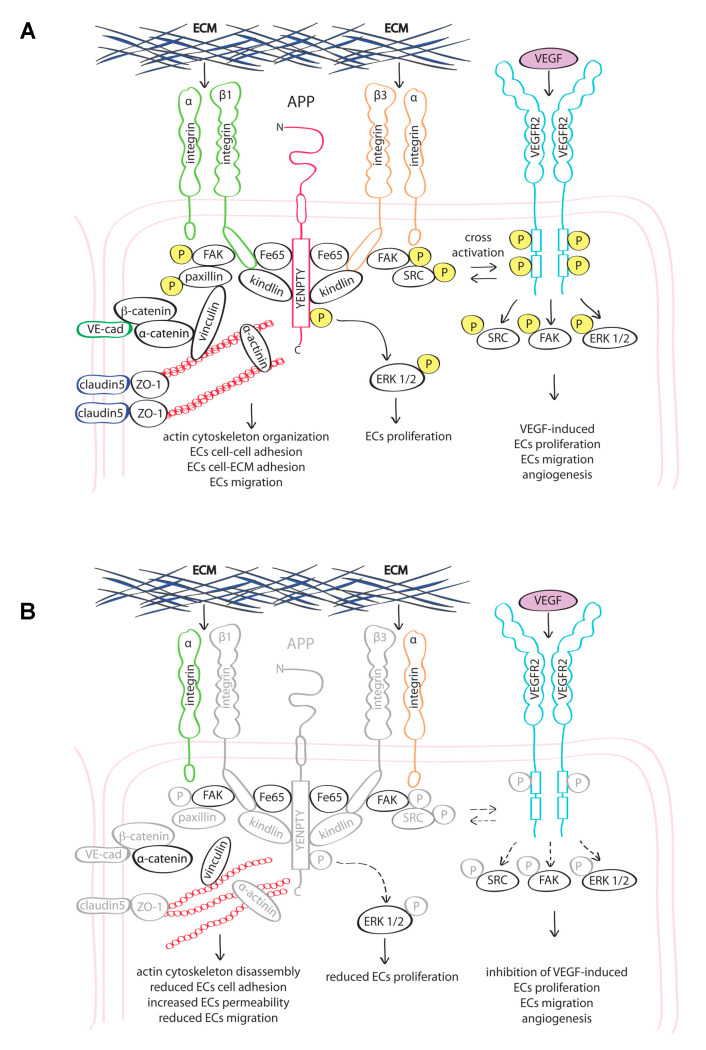
Proposed working model: (**A**) APP interacts with the integrin β-subunit. This interaction promotes the stability and functionality of focal adhesions and their correct localization at the cell membrane, and activation of VEGFR2; (**B**) Loss of APP leads to an altered expression and activation of actin cytoskeleton-interacting proteins (represented in grey) and to the reduced activity of several proteins at the cell membrane, included VEGFR2, ultimately causing endothelial dysfunction. Abbreviations: ECM = extracellular matrix; (P) = phosphorylation site.

Taken together, our results indicate that APP is necessary for the focal adhesion complex expression/stability. The interaction with cytoskeleton-interacting proteins such integrins, is essential to maintain ECs proliferation, adhesion and angiogenesis. Loss of APP expression results in altered cellular response to environmental stimuli and leads to endothelial dysfunction (Figure 7).

## 5. Conclusions

While the pathological outcome of APP dysregulation is mainly associated to its overexpression, recent studies showed that APP expression levels tend to decrease with age [59,60], and APP-knockout mice show age-dependent cognitive deficit and impaired locomotor activity [61,62]. Moreover, senescent brains showed an increase of amyloidogenic processing mediated by BACE1 [63], indicating that amyloid-β peptides accumulation is not necessarily associated with APP overexpression. Furthermore, clinical trials targeting amyloid-β have failed to reverse the cognitive loss, and in some cases resulted in vascular severe adverse events (i.e., bapineuzumab), indicating the importance of physiological levels of amyloid-β peptides and highlighting the urgency to better understand the function of APP and its cleavage products in cell homeostasis.

In this study, we show that a correct expression level of APP may be necessary for the correct functionality of ECs. Although the use of HUVEC as model is not representative of all endothelial cell types found in an organism, they are an excellent model for the study of vascular endothelium properties and the main biological pathways involved in endothelium function. More studies addressing the APP function specifically in cerebrovascular endothelium will be needed. 

A deep understanding of APP function in maintaining vascular homeostasis might shed light on new therapeutic targets and provide a new perspective on treatment options of neurodegenerative diseases.

## Supplementary Materials

The following are available online at https://www.mdpi.com/2073-4409/9/11/2506/s1, Figure S1: Selection of APP targeting siRNAs. Figure S2: Label free proteomic analysis of HUVEC following APP knockdown. Figure S3: APP doesn’t physically interact with VEGFR2 and APP knockdown doesn’t reduce mRNA levels of VEGFR2-VEGF downstream signaling. Figure S4: Western Blot analysis with alternative antibodies. Table S1: List of up-regulated and down-regulated proteins.
