# Supplementary material for: Amyloid-β Precursor Protein APP Down-Regulation Alters Actin Cytoskeleton-Interacting Proteins in Endothelial Cells
Source: Cells. 2020 Nov 19;9(11):2506. doi: 10.3390/cells9112506 (PMC7699411; doi:10.3390/cells9112506)
Supplement: Supplementary file 1 [file cells-09-02506-s001.pdf]

# Amyloid-β Precursor Protein APP down-regulation alters actin cytoskeleton-interacting proteins in endothelial cells.

**Emma Ristori [1,2], Vittoria Cicaloni [2], Laura Salvini [2], Laura Tinti [2], Cristina Tinti [2], Michael Simons [3,4], Federico Corti [3], Sandra Donnini* [1,2] and Marina Ziche* [2,5]**

**Supplementary Materials:**

- Figure S1: Selection of APP targeting siRNAs.
- Figure S2: Label free proteomic analysis of HUVEC following APP knockdown.
- Figure S3: APP doesn't physically interact with VEGFR2 and APP knockdown doesn't reduce mRNA levels of VEGFR2-VEGF downstream signaling.
- Figure S4: Western blot analysis with alternative antibodies.
- Table S1: List of up-regulated and down-regulated proteins.

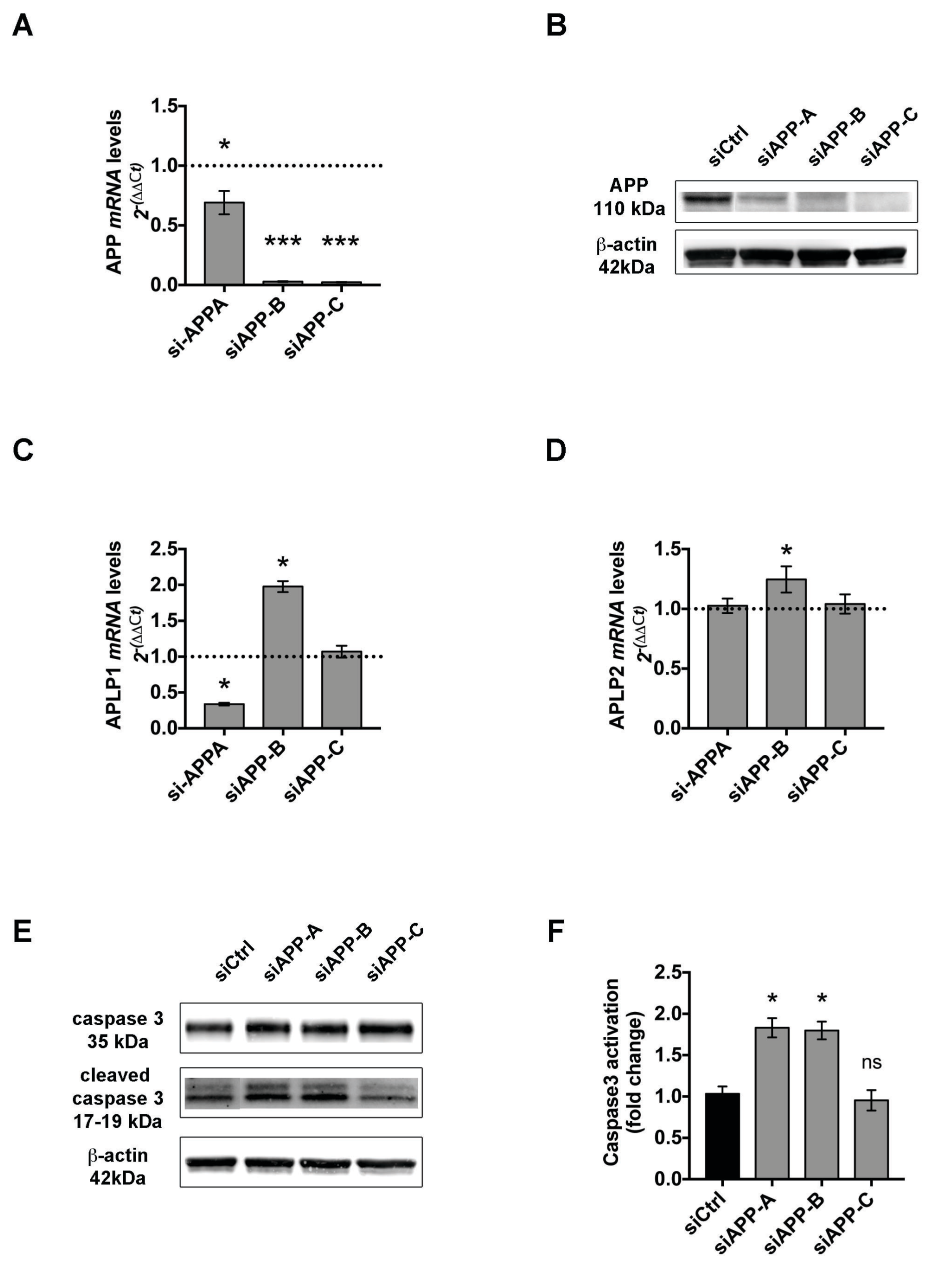


**Figure S1**. Selection of APP targeting siRNAs: **(a)** RTqPCR showing APP mRNA expression levels upon silencing for 48 hours with siAPP-A, -B or -C respectively; **(b)** Western blot showing reduced protein expression levels of APP upon silencing for 48 hours with siAPP-A, -B or -C respectively; **(c-d)** RTqPCR showing mRNA expression levels of APLP1 (c) and APLP2 (d). siAPP-C doesn't affect APLP1 and APLP2 expression. On the contrary, siAPP-A and siAPP-B show an aspecific off-target effect on APLP1 and APLP2; **(e)** Western-blot showing activation of apoptotic Caspase-3 in silenced cells (siAPP) and control (siCtrl). Activation of Caspase-3 was measured as cleaved Caspase-3/total Caspase3 ratio expression; **(f)** Quantification of western blot analysis (n=3) in (e) shows that siAPP-C doesn't promote apoptosis in silenced cells, whereas Caspase-3 is significantly activated by siAPP-A and siAPP-B. All data are presented as mean ± SEM, n=3 replicates, *P<0.05, ***P<0.001.

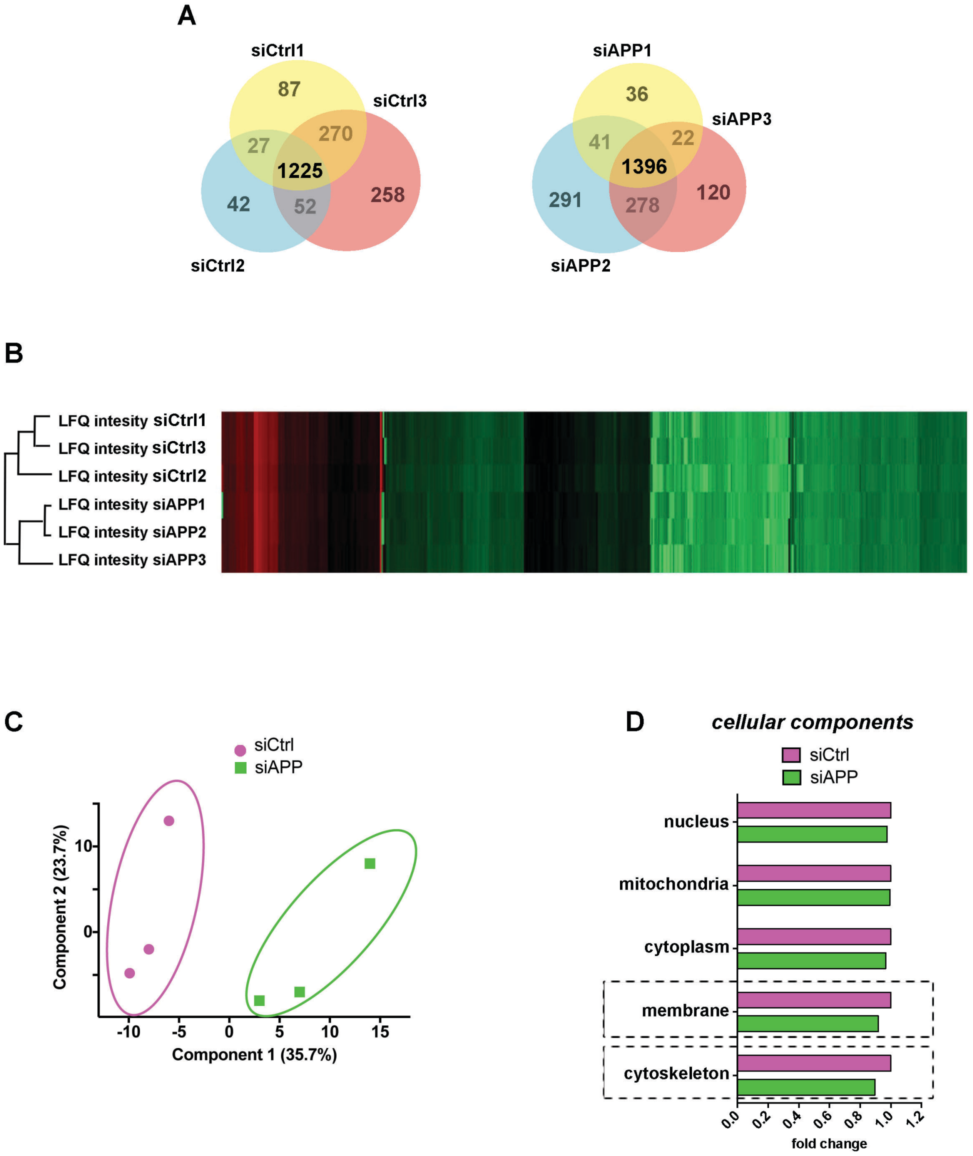


**Figure S2.** Label free proteomic analysis of HUVEC following APP knockdown: **(a)** Venn diagrams representing the number of reproducibly quantified proteins from three biological replicates of the HUVEC control and HUVEC silenced for APP for 48 hours. The common protein ensembles amount to 1225 and 1396 for siCrl and siAPP respectively; **(b)** Hierarchical clustering. Heatmap shows LFQ intensity values for triplicates of every sample. Within each group, proteins are sorted according to their LFQ intensity values: red (max value), green (min value). Clustering tree resulting from unsupervised clustering of samples is shown on the left and confirms a differentiation between siCtrl and siAPP samples; **(c)** PCA-biplot showing a principal component analysis of all siCtrl and siAPP samples analyzed by mass spectrometry. All the triplicates of siCtrl and siAPP samples appeared to cluster in two distinct groups confirming a clear proteomic differentiation between the two datasets. The percentage of the variance contributed by each principal component is indicated in the axis: 35,7% for the first component and 23.7% for the second component; **(d)** Functional comparison between siCtrl common dataset and siAPP common dataset was performed to analyze differences (p-value < 0.001) in cellular components.

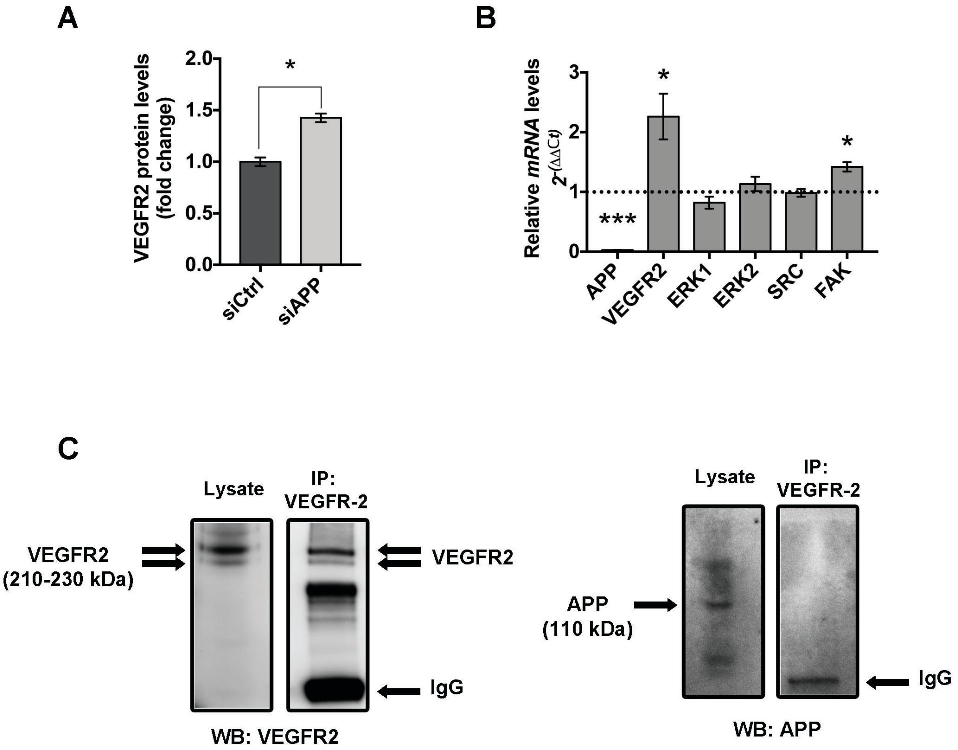


**Figure S3.** APP doesn't physically interact with VEGFR2 and APP knockdown doesn’t reduce mRNA levels of VEGFR2-VEGF downstream signaling **(a)** Western blot analysis of total VEGFR2 protein expression in siAPP HUVEC **(b)** RTqPCR analysis of VEGFR2 and downstream signaling mRNA levels **(c)** Immunoprecipitation (IP) was performed using anti-VEGFR2 antibody (IP: anti-VEGFR2), followed by western blot analysis to check the presence of APP in the pulled-down complex (WB). The presence of VEGFR2 in the immunoprecipitate was used as positive control. Whole cell lysates (Lysate) were also analyzed for total VEGFR2 and APP protein level. The pulled down of anti-VEGFR2 antibody showed that it was able to bind the VEGFR2 protein but not APP, suggesting that the two proteins don't physically interact.

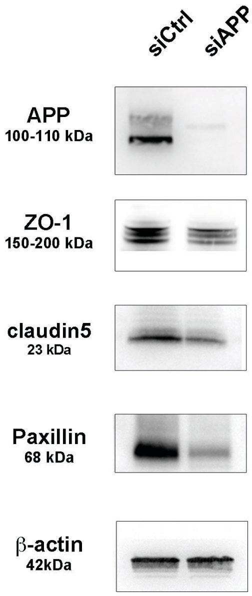


**Figure S4**: Western Blot analysis with alternative antibodies. ZO-1, Claudin5 and paxillin protein expression was assessed with different antibodies. Detection of ZO-1 with Life Technologies antibody (#61-7300), gives comparable results to Thermo Fisher Scientific antibody reported on the main text (# 33-9100). Similarly, protein expression levels resulted reduced in siAPP HUVEC using anti-claudin5 antibody from Thermo Fisher Scientific (#35-250) and anti-paxillin antibody from Abcam (#ab32084), and this result is comparable to the blots reported in the main text and obtained using respectively anti-claudin5 from Abcam (#ab53765), and anti-paxillin from Millipore (#3794).

**Table S1: List of up-regulated and down-regulated proteins.**

| Significant | -LOG(p-value) | Uniprot Accession | Uniprot ID | Name | Species | Gene | Up/down-regulation |
|---|---|---|---|---|---|---|---|
| + | 1.696933953 | A0AVT1 | UBA6_HUMAN | Ubiquitin-likemodifier-activatingenzyme6 | Homo sapiens | UBA6 | up-regulated |
| + | 2.522674384 | A5A6H4 | ROA1_HUMAN | HeterogeneousnuclearribonucleoproteinA1 | Homo sapiens | HNRNPA1 | down-regulated |
| + | 1.706431017 | O00151 | PDLI1_HUMAN | PDZandLIMdomainprotein1 | Homo sapiens | PDLIM1 | down-regulated |
| + | 2.090178398 | O00159 | MYO1C_HUMAN | Unconventionalmyosin-Ic | Homo sapiens | MYO1C | down-regulated |
| + | 3.17838226 | O00469 | PLOD2_HUMAN | Procollagen-lysine,2-oxoglutarate5-dioxygenase2 | Homo sapiens | PLOD2 | down-regulated |
| + | 2.743386048 | O00571 | DDX3X_HUMAN | ATP-dependentRNAhelicaseDDX3X | Homo sapiens | DDX3X | down-regulated |
| + | 2.750845176 | O00625 | PIR_HUMAN | Pirin | Homo sapiens | PIR | down-regulated |
| + | 1.514993551 | O00767 | ACOD_HUMAN | Acyl-CoAdesaturase | Homo sapiens | SCD | down-regulated |
| + | 1.160610103 | O14561 | ACPM_HUMAN | Acylcarrierprotein,mitochondrial | Homo sapiens | NDUFAB1 | down-regulated |
| + | 3.687649448 | O14879 | IFIT3_HUMAN | Interferon-inducedproteinwithtetratricopeptiderepeats3 | Homo sapiens | IFIT3 | up-regulated |
| + | 2.015502669 | O14933 | UB2L6_HUMAN | Ubiquitin/ISG15-conjugatingenzymeE2L6 | Homo sapiens | UBE2L6 | up-regulated |
| + | 2.440057636 | O15173 | PGRC2_HUMAN | Membrane-associatedprogesteronereceptorcomponent2 | Homo sapiens | PGRMC2 | up-regulated |
| + | 1.054930824 | O15460 | P4HA2_HUMAN | Prolyl4-hydroxylasesubunitalpha-2 | Homo sapiens | P4HA2 | up-regulated |
| + | 1.757811053 | O15533 | TPSN_HUMAN | Tapasin | Homo sapiens | TAPBP | up-regulated |
| + | 1.387369479 | O75352 | MPU1_HUMAN | Mannose-P-dolicholutilizationdefect1protein | Homo sapiens | MPDU1 | up-regulated |
| + | 2.236427975 | O75964 | ATP5L_HUMAN | ATPsynthasesubunitg,mitochondrial | Homo sapiens | ATP5MG | down-regulated |
| + | 1.899778643 | O94760 | DDAH1_HUMAN | N(G),N(G)-dimethylargininedimethylaminohydrolase1 | Homo sapiens | DDAH1 | up-regulated |
| + | 1.765031675 | O95340 | PAPS2_HUMAN | Bifunctional3'-phosphoadenosine5'-phosphosulfatesynthase2 | Homo sapiens | PAPSS2 | up-regulated |
| + | 1.53277814 | O95394 | AGM1_HUMAN | Phosphoacetylglucosaminemutase | Homo sapiens | PGM3 | up-regulated |
| + | 2.534386104 | O95786 | DDX58_HUMAN | ProbableATP-dependentRNAhelicaseDDX58 | Homo sapiens | DDX58 | up-regulated |
| + | 1.114553442 | O95864 | FADS2_HUMAN | Acyl-CoA6-desaturase | Homo sapiens | FADS2 | down-regulated |
| + | 2.052784401 | P00352 | AL1A1_HUMAN | Retinaldehydrogenase1 | Homo sapiens | ALDH1A1 | down-regulated |
| + | 2.160872861 | P02751 | FINC_HUMAN | Fibronectin | Homo sapiens | FN1 | up-regulated |
| + | 1.440434415 | P00973 | OAS1_HUMAN | 2'-5'-oligoadenylatesynthase1 | Homo sapiens | OAS1 | up-regulated |
| + | 2.251688608 | P04406 | G3P_HUMAN | Glyceraldehyde-3-phosphatedehydrogenase | Homo sapiens | GAPDH | down-regulated |
| + | 2.717914577 | P05121 | PAI1_HUMAN | Plasminogenactivatorinhibitor1 | Homo sapiens | SERPINE1 | up-regulated |
| + | 1.603052806 | P05556 | ITB1_HUMAN | Integrinbeta-1 | Homo sapiens | ITGB1 | down-regulated |
| + | 1.526031727 | P07737 | PROF1_HUMAN | Profilin-1 | Homo sapiens | PFN1 | down-regulated |
| + | 2.518157148 | P05161 | ISG15_HUMAN | Ubiquitin-likeproteinISG15 | Homo sapiens | ISG15 | up-regulated |
| + | 3.119174198 | P05362 | ICAM1_HUMAN | Intercellularadhesionmolecule1 | Homo sapiens | ICAM1 | up-regulated |
| + | 2.209848209 | P07996 | TSP1_HUMAN | Thrombospondin-1 | Homo sapiens | THBS1 | down-regulated |
| + | 1.242351574 | P06703 | S10A6_HUMAN | ProteinS100-A6 | Homo sapiens | S100A6 | down-regulated |
| + | 2.25465283 | P06744 | G6PI_HUMAN | Glucose-6-phosphateisomerase | Homo sapiens | GPI | down-regulated |
| + | 1.855508392 | P09211 | GSTP1_HUMAN | GlutathioneS-transferaseP | Homo sapiens | GSTP1 | up-regulated |

| ignificant | -LOG(p-value) | Uniprot Accession | Uniprot ID | Name | Species | Gene | Up/down-regulation |
|---|---|---|---|---|---|---|---|
| + | 1.740222305 | P09382 | LEG1_HUMAN | Galectin-1 | Homo sapiens | LGALS1 | down-regulated |
| + | 2.368520131 | P08195 | 4F2_HUMAN | 4F2cell-surfaceantigenheavychain | Homo sapiens | SLC3A2 | down-regulated |
| + | 1.891527327 | P09493 | TPM1_HUMAN | Tropomyosinalpha-1chain | Homo sapiens | TPM1 | up-regulated |
| + | 1.443180756 | P09874 | PARP1_HUMAN | Poly[ADP-ribose]polymerase1 | Homo sapiens | PARP1 | down-regulated |
| + | 3.032771209 | P12814 | ACTN1_HUMAN | Alpha-actinin-1 | Homo sapiens | ACTN1 | down-regulated |
| + | 2.692592448 | P09525 | ANXA4_HUMAN | AnnexinA4 | Homo sapiens | ANXA4 | down-regulated |
| + | 1.897264404 | P09601 | HMOX1_HUMAN | Hemeoxygenase1 | Homo sapiens | HMOX1 | down-regulated |
| + | 2.727149711 | P14550 | AK1A1_HUMAN | Aldo-ketoreductasefamily1memberA1 | Homo sapiens | AKR1A1 | down-regulated |
| + | 2.790370236 | P09914 | IFIT1_HUMAN | Interferon-inducedproteinwithtetratricopeptiderepeats1 | Homo sapiens | IFIT1 | up-regulated |
| + | 1.609073654 | Q6PI52 | CALM_HUMAN | Calmodulin | Homo sapiens | CALM3 | down-regulated |
| + | 3.095542821 | P10644 | KAP0_HUMAN | cAMP-dependentproteinkinasetypeI-alpharegulatorysubunit | Homo sapiens | PRKAR1A | down-regulated |
| + | 3.620163005 | P11413 | G6PD_HUMAN | Glucose-6-phosphate1-dehydrogenase | Homo sapiens | G6PD | down-regulated |
| + | 2.40258352 | P11586 | C1TC_HUMAN | C-1-tetrahydrofolatesynthase,cytoplasmic | Homo sapiens | MTHFD1 | down-regulated |
| + | 4.625454001 | P12268 | IMDH2_HUMAN | Inosine-5'-monophosphatedehydrogenase2 | Homo sapiens | IMPDH2 | down-regulated |
| + | 1.894706253 | P15121 | ALDR_HUMAN | Aldo-ketoreductasefamily1memberB1 | Homo sapiens | AKR1B1 | down-regulated |
| + | 2.581743903 | P13164 | IFM1_HUMAN | Interferon-inducedtransmembraneprotein1 | Homo sapiens | IFITM1 | up-regulated |
| + | 2.327212877 | P13473 | LAMP2_HUMAN | Lysosome-associatedmembraneglycoprotein2 | Homo sapiens | LAMP2 | down-regulated |
| + | 3.263787615 | P13598 | ICAM2_HUMAN | Intercellularadhesionmolecule2 | Homo sapiens | ICAM2 | down-regulated |
| + | 1.564748621 | P14174 | MIF_HUMAN | Macrophagemigrationinhibitoryfactor | Homo sapiens | MIF | down-regulated |
| + | 2.989539155 | P14317 | HCLS1_HUMAN | Hematopoieticlineagecell-specificprotein | Homo sapiens | HCLS1 | up-regulated |
| + | 2.910071374 | P35241 | RADI_HUMAN | Radixin | Homo sapiens | RDX | down-regulated |
| + | 3.191404542 | P48681 | NEST_HUMAN | Nestin | Homo sapiens | NES | down-regulated |
| + | 3.559212247 | P15144 | AMPN_HUMAN | AminopeptidaseN | Homo sapiens | ANPEP | down-regulated |
| + | 1.415200052 | P15559 | NQO1_HUMAN | NAD(P)Hdehydrogenase[quinone]1 | Homo sapiens | NQO1 | down-regulated |
| + | 3.442546508 | P16949 | STMN1_HUMAN | Stathmin | Homo sapiens | STMN1 | down-regulated |
| + | 1.901770836 | P17301 | ITA2_HUMAN | Integrinalpha-2 | Homo sapiens | ITGA2 | down-regulated |
| + | 1.640455436 | P17612 | KAPCA_HUMAN | cAMP-dependentproteinkinasecatalyticsubunitalpha | Homo sapiens | PRKACA | down-regulated |
| + | 2.284685181 | P17655 | CAN2_HUMAN | Calpain-2catalyticsubunit | Homo sapiens | CAPN2 | down-regulated |
| + | 3.048530771 | P19971 | TYPH_HUMAN | Thymidinephosphorylase | Homo sapiens | TYMP | up-regulated |
| + | 3.336400095 | P20591 | MX1_HUMAN | Interferon-inducedGTP-bindingproteinMx1 | Homo sapiens | MX1 | up-regulated |
| + | 2.287577839 | P20700 | LMNB1_HUMAN | Lamin-B1 | Homo sapiens | LMNB1 | down-regulated |
| + | 1.857054822 | P21589 | 5NTD_HUMAN | 5'-nucleotidase | Homo sapiens | NT5E | down-regulated |
| + | 1.982435862 | P21964 | COMT_HUMAN | CatecholO-methyltransferase | Homo sapiens | COMT | up-regulated |
| + | 1.757846938 | P22626 | ROA2_HUMAN | HeterogeneousnuclearribonucleoproteinsA2/B1 | Homo sapiens | HNRNPA2B1 | up-regulated |

| Significant | -LOG(p-value) | Uniprot Accession | Uniprot ID | Name | Species | Gene | Up/down-regulation |
|---|---|---|---|---|---|---|---|
| + | 1.722048997 | P23497 | SP100_HUMAN | NuclearautoantigenSp-100 | Homo sapiens | SP100 | up-regulated |
| + | 2.81892956 | P25205 | MCM3_HUMAN | DNAreplicationlicensingfactorMCM3 | Homo sapiens | MCM3 | down-regulated |
| + | 1.347814439 | P26640 | SYVC_HUMAN | Valine--tRNAligase | Homo sapiens | VARS | down-regulated |
| + | 1.171753598 | P49419 | AL7A1_HUMAN | Alpha-aminoadipicsemialdehydedehydrogenase | Homo sapiens | ALDH7A1 | down-regulated |
| + | 1.346818553 | P29466 | CASP1_HUMAN | Caspase-1 | Homo sapiens | CASP1 | up-regulated |
| + | 1.638845632 | P29590 | PML_HUMAN | ProteinPML | Homo sapiens | PML | up-regulated |
| + | 1.43416805 | P30043 | BLVRB_HUMAN | Flavinreductase(NADPH) | Homo sapiens | BLVRB | up-regulated |
| + | 2.540767736 | P30837 | AL1B1_HUMAN | AldehydedehydrogenaseX,mitochondrial | Homo sapiens | ALDH1B1 | down-regulated |
| + | 2.795995598 | P32455 | GBP1_HUMAN | Guanylate-bindingprotein1 | Homo sapiens | GBP1 | up-regulated |
| + | 2.464128618 | P33316 | DUT_HUMAN | Deoxyuridine5'-triphosphatenucleotidohydrolase,mitochondrial | Homo sapiens | DUT | down-regulated |
| + | 2.558422427 | P33991 | MCM4_HUMAN | DNAreplicationlicensingfactorMCM4 | Homo sapiens | MCM4 | down-regulated |
| + | 2.884703964 | P33992 | MCM5_HUMAN | DNAreplicationlicensingfactorMCM5 | Homo sapiens | MCM5 | down-regulated |
| + | 2.754910472 | P33993 | MCM7_HUMAN | DNAreplicationlicensingfactorMCM7 | Homo sapiens | MCM7 | down-regulated |
| + | 1.604719285 | P52943 | CRIP2_HUMAN | Cysteine-richprotein2 | Homo sapiens | CRIP2 | down-regulated |
| + | 1.333266232 | P37268 | FDFT_HUMAN | Squalenesynthase | Homo sapiens | FDFT1 | down-regulated |
| + | 1.212427275 | P41226 | UBA7_HUMAN | Ubiquitin-likemodifier-activatingenzyme7 | Homo sapiens | UBA7 | up-regulated |
| + | 1.821453521 | P42224 | STAT1_HUMAN | Signaltransducerandactivatoroftranscription1-alpha/beta | Homo sapiens | STAT1 | up-regulated |
| + | 2.449511919 | P42892 | ECE1_HUMAN | Endothelin-convertingenzyme1 | Homo sapiens | ECE1 | down-regulated |
| + | 2.298576947 | P43121 | MUC18_HUMAN | CellsurfaceglycoproteinMUC18 | Homo sapiens | MCAM | down-regulated |
| + | 1.679473775 | P43246 | MSH2_HUMAN | DNAmismatchrepairproteinMsh2 | Homo sapiens | MSH2 | down-regulated |
| + | 1.861651687 | P56556 | NDUA6_HUMAN | NADHdehydrogenase[ubiquinone]1alphasubcomplexsubunit6 | Homo sapiens | NDUFA6 | down-regulated |
| + | 2.200140577 | P48735 | IDHP_HUMAN | Isocitratedehydrogenase[NADP],mitochondrial | Homo sapiens | IDH2 | down-regulated |
| + | 1.593918793 | P68366 | TBA4A_HUMAN | Tubulinalpha-4Achain | Homo sapiens | TUBA4A | down-regulated |
| + | 1.597223282 | P49593 | PPM1F_HUMAN | Proteinphosphatase1F | Homo sapiens | PPM1F | down-regulated |
| + | 1.49838734 | P49736 | MCM2_HUMAN | DNAreplicationlicensingfactorMCM2 | Homo sapiens | MCM2 | down-regulated |
| + | 2.07882652 | Q10739 | MMP14_HUMAN | Matrixmetalloproteinase-14 | Homo sapiens | MMP14 | down-regulated |
| + | 2.609896121 | P50453 | SPB9_HUMAN | SerpinB9 | Homo sapiens | SERPINB9 | up-regulated |
| + | 2.514183214 | P98160 | PGBM_HUMAN | Basementmembrane-specificheparansulfateproteoglycancoreprotein | Homo sapiens | HSPG2 | down-regulated |
| + | 2.274894235 | P51991 | ROA3_HUMAN | HeterogeneousnuclearribonucleoproteinA3 | Homo sapiens | HNRNPA3 | up-regulated |
| + | 1.611304258 | P52306 | GDS1_HUMAN | Rap1GTPase-GDPdissociationstimulator1 | Homo sapiens | RAP1GDS1 | down-regulated |
| + | 2.321143717 | P52597 | HNRPF_HUMAN | HeterogeneousnuclearribonucleoproteinF | Homo sapiens | HNRNPF | down-regulated |
| + | 1.329352619 | Q03252 | LMNB2_HUMAN | Lamin-B2 | Homo sapiens | LMNB2 | down-regulated |
| + | 1.751193241 | P53999 | TCP4_HUMAN | ActivatedRNApolymeraseIItranscriptionalcoactivatorp15 | Homo sapiens | SUB1 | up-regulated |

| Significant | -LOG(p-value) | Uniprot Accession | Uniprot ID | Name | Species | Gene | Up/down-regulation |
|---|---|---|---|---|---|---|---|
| + | 2.646805136 | Q09666 | AHNK_HUMAN | Neuroblastdifferentiation-associatedproteinAHNAK | Homo sapiens | AHNAK | down-regulated |
| + | 1.729091791 | P60903 | S10AA_HUMAN | ProteinS100-A10 | Homo sapiens | S100A10 | down-regulated |
| + | 1.942369382 | P62805 | H4_HUMAN | HistoneH4 | Homo sapiens | HIST1H4A | down-regulated |
| + | 2.10781878 | P62873 | GBB1_HUMAN | Guaninenucleotide-bindingproteinG(I)/G(S)/G(T)subunitbeta-1 | Homo sapiens | GNB1 | down-regulated |
| + | 2.121541309 | Q13045 | FLII_HUMAN | Proteinflightless-1homolog | Homo sapiens | FLII | down-regulated |
| + | 1.752420731 | Q71DI3 | H32_HUMAN | HistoneH3.2 | Homo sapiens | HIST2H3A | down-regulated |
| + | 1.247860179 | P80217 | IN35_HUMAN | Interferon-induced35kDaprotein | Homo sapiens | IFI35 | up-regulated |
| + | 2.580596148 | Q14315 | FLNC_HUMAN | Filamin-C | Homo sapiens | FLNC | down-regulated |
| + | 1.878338705 | Q01813 | PFKAP_HUMAN | ATP-dependent6-phosphofructokinase,platelettype | Homo sapiens | PFKP | down-regulated |
| + | 2.280218766 | Q01995 | TAGL_HUMAN | Transgelin | Homo sapiens | TAGLN | down-regulated |
| + | 2.452618714 | Q02790 | FKBP4_HUMAN | Peptidyl-prolylcis-transisomeraseFKBP4 | Homo sapiens | FKBP4 | up-regulated |
| + | 2.102109949 | Q15019 | SEPT2_HUMAN | Septin-2 | Homo sapiens | SEPT2 | down-regulated |
| + | 2.315430492 | Q03518 | TAP1_HUMAN | Antigenpeptidetransporter1 | Homo sapiens | TAP1 | up-regulated |
| + | 1.363322983 | Q05519 | SRS11_HUMAN | Serine/arginine-richsplicingfactor11 | Homo sapiens | SRSF11 | down-regulated |
| + | 2.233465532 | Q06210 | GFPT1_HUMAN | Glutamine--fructose-6-phosphateaminotransferase[isomerizing]1 | Homo sapiens | GFPT1 | up-regulated |
| + | 2.236564905 | Q15149 | PLEC_HUMAN | Plectin | Homo sapiens | PLEC | down-regulated |
| + | 1.549274675 | Q10471 | GALT2_HUMAN | PolypeptideN-acetylgalactosaminyltransferase2 | Homo sapiens | GALNT2 | down-regulated |
| + | 1.320606115 | Q10472 | GALT1_HUMAN | PolypeptideN-acetylgalactosaminyltransferase1 | Homo sapiens | GALNT1 | up-regulated |
| + | 2.260549256 | Q12874 | SF3A3_HUMAN | Splicingfactor3Asubunit3 | Homo sapiens | SF3A3 | down-regulated |
| + | 1.201522635 | Q13011 | ECH1_HUMAN | Delta(3,5)-Delta(2,4)-dienoyl-CoAisomerase,mitochondrial | Homo sapiens | ECH1 | down-regulated |
| + | 1.628826877 | Q16181 | SEPT7_HUMAN | Septin-7 | Homo sapiens | SEPT7 | down-regulated |
| + | 1.354861439 | Q13451 | FKBP5_HUMAN | Peptidyl-prolylcis-transisomeraseFKBP5 | Homo sapiens | FKBP5 | down-regulated |
| + | 2.102855785 | Q13619 | CUL4A_HUMAN | Cullin-4A | Homo sapiens | CUL4A | down-regulated |
| + | 1.878398865 | Q14108 | SCRB2_HUMAN | Lysosomemembraneprotein2 | Homo sapiens | SCARB2 | up-regulated |
| + | 1.549515149 | Q16555 | DPYL2_HUMAN | Dihydropyrimidinase-relatedprotein2 | Homo sapiens | DPYSL2 | down-regulated |
| + | 3.398949891 | Q14554 | PDIA5_HUMAN | Proteindisulfide-isomeraseA5 | Homo sapiens | PDIA5 | down-regulated |
| + | 2.4553328 | Q14764 | MVP_HUMAN | Majorvaultprotein | Homo sapiens | MVP | down-regulated |
| + | 2.324005502 | Q14914 | PTGR1_HUMAN | Prostaglandinreductase1 | Homo sapiens | PTGR1 | down-regulated |
| + | 3.406335429 | Q16658 | FSCN1_HUMAN | Fascin | Homo sapiens | FSCN1 | down-regulated |
| + | 2.302852662 | Q99439 | CNN2_HUMAN | Calponin-2 | Homo sapiens | CNN2 | up-regulated |
| + | 3.283138273 | Q15646 | OASL_HUMAN | 2'-5'-oligoadenylatesynthase-likeprotein | Homo sapiens | OASL | up-regulated |
| + | 2.217394578 | Q9BUF5 | TBB6_HUMAN | Tubulinbeta-6chain | Homo sapiens | TUBB6 | down-regulated |
| + | 2.561061429 | Q9NZN4 | EHD2_HUMAN | EHdomain-containingprotein2 | Homo sapiens | EHD2 | down-regulated |
| + | 1.577606286 | Q9ULV4 | COR1C_HUMAN | Coronin-1C | Homo sapiens | CORO1C | down-regulated |

| Significant | -LOG(p-value) | Uniprot Accession | Uniprot ID | Name | Species | Gene | Up/down-regulation |
|---|---|---|---|---|---|---|---|
| + | 1.999767899 | Q16836 | HCDH_HUMAN | Hydroxyacyl-coenzymeAdehydrogenase,mitochondrial | Homo sapiens | HADH | down-regulated |
| + | 1.826012273 | Q53EP0 | FND3B_HUMAN | FibronectintypeIIIdomain-containingprotein3B | Homo sapiens | FNDC3B | up-regulated |
| + | 1.552276205 | Q58FF8 | H90B2_HUMAN | PutativeheatshockproteinHSP90-beta2 | Homo sapiens | HSP90AB2P | up-regulated |
| + | 1.270364733 | Q5EBM0 | CMPK2_HUMAN | UMP-CMPkinase2,mitochondrial | Homo sapiens | CMPK2 | up-regulated |
| + | 1.463361821 | Q5U651 | RAIN_HUMAN | Ras-interactingprotein1 | Homo sapiens | RASIP1 | down-regulated |
| + | 1.787507794 | Q9C0H2 | TTYH3_HUMAN | Proteintweetyhomolog3 | Homo sapiens | TTYH3 | down-regulated |
| + | 1.650812059 | Q6P5R6 | RL22L_HUMAN | 60SribosomalproteinL22-like1 | Homo sapiens | RPL22L1 | down-regulated |
| + | 2.039280783 | Q7Z2W4 | ZCCHV_HUMAN | ZincfingerCCCH-typeantiviralprotein1 | Homo sapiens | ZC3HAV1 | up-regulated |
| + | 1.878171649 | Q86UX7 | URP2_HUMAN | Fermitinfamilyhomolog3 | Homo sapiens | FERMT3 | down-regulated |
| + | 1.583697895 | Q8NC42 | RN149_HUMAN | E3ubiquitin-proteinligaseRNF149 | Homo sapiens | RNF149 | down-regulated |
| + | 1.497383781 | Q8WWM7 | ATX2L_HUMAN | Ataxin-2-likeprotein | Homo sapiens | ATXN2L | up-regulated |
| + | 1.694896568 | Q92890 | UFD1_HUMAN | UbiquitinrecognitionfactorinER-associateddegradationprotein1 | Homo sapiens | UFD1 | up-regulated |
| + | 1.969505466 | Q969H8 | MYDGF_HUMAN | Myeloid-derivedgrowthfactor | Homo sapiens | MYDGF | up-regulated |
| + | 1.382502901 | Q96C19 | EFHD2_HUMAN | EF-handdomain-containingproteinD2 | Homo sapiens | EFHD2 | down-regulated |
| + | 1.542673009 | Q96CV9 | OPTN_HUMAN | Optineurin | Homo sapiens | OPTN | up-regulated |
| + | 1.613865805 | Q96HY6 | DDRGK_HUMAN | DDRGKdomain-containingprotein1 | Homo sapiens | DDRGK1 | up-regulated |
| + | 2.875678045 | Q96IZ0 | PAWR_HUMAN | PRKCapoptosisWT1regulatorprotein | Homo sapiens | PAWR | up-regulated |
| + | 1.211081218 | Q96JJ7 | TMX3_HUMAN | Proteindisulfide-isomeraseTMX3 | Homo sapiens | TMX3 | up-regulated |
| + | 1.801323121 | Q96S97 | MYADM_HUMAN | Myeloid-associateddifferentiationmarker | Homo sapiens | MYADM | up-regulated |
| + | 4.192993681 | P00441 | SODC_HUMAN | Superoxidedismutase[Cu-Zn] | Homo sapiens | SOD1 | up-regulated |
| + | 1.392978492 | Q99584 | S10AD_HUMAN | ProteinS100-A13 | Homo sapiens | S100A13 | up-regulated |
| + | 1.63392292 | Q9BRX8 | PXL2A_HUMAN | Peroxiredoxin-like2A | Homo sapiens | PRXL2A | down-regulated |
| + | 1.948658409 | Q9BS26 | ERP44_HUMAN | Endoplasmicreticulumresidentprotein44 | Homo sapiens | ERP44 | up-regulated |
| + | 1.617333177 | P04275 | VWF_HUMAN | vonWillebrandfactor | Homo sapiens | VWF | down-regulated |
| + | 1.766838223 | Q9H173 | SIL1_HUMAN | NucleotideexchangefactorSIL1 | Homo sapiens | SIL1 | up-regulated |
| + | 1.943140258 | Q9NTK5 | OLA1_HUMAN | Obg-likeATPase1 | Homo sapiens | OLA1 | down-regulated |
| + | 1.830215639 | Q9NUL5 | RYDEN_HUMAN | RepressorofyieldofDENVprotein | Homo sapiens | RYDEN | up-regulated |
| + | 0.842053064 | Q9NY15 | STAB1_HUMAN | Stabilin-1 | Homo sapiens | STAB1 | up-regulated |
| + | 2.598572211 | Q9NYL4 | FKB11_HUMAN | Peptidyl-prolylcis-transisomeraseFKBP11 | Homo sapiens | FKBP11 | up-regulated |
| + | 1.329038421 | Q9NZ45 | CISD1_HUMAN | CDGSHiron-sulfurdomain-containingprotein1 | Homo sapiens | CISD1 | up-regulated |
| + | 2.438077482 | Q9NZM1 | MYOF_HUMAN | Myoferlin | Homo sapiens | MYOF | down-regulated |
| + | 1.548401269 | P29279 | CCN2_HUMAN | CCNfamilymember2 | Homo sapiens | CCN2 | up-regulated |
| + | 1.789612702 | Q9UBS4 | DJB11_HUMAN | DnaJhomologsubfamilyBmember11 | Homo sapiens | DNAJB11 | up-regulated |
| + | 1.932532493 | Q9UKK3 | PARP4_HUMAN | Proteinmono-ADP-ribosyltransferasePARP4 | Homo sapiens | PARP4 | down-regulated |

| Significant | -LOG(p-value) | Uniprot Accession | Uniprot ID | Name | Species | Gene | Up/down-regulation |
|---|---|---|---|---|---|---|---|
| + | 2.121041419 | P50570 | DYN2_HUMAN | Dynamin-2 | Homo sapiens | DNM2 | up-regulated |
| + | 1.663875263 | Q9Y371 | SHLB1_HUMAN | Endophilin-B1 | Homo sapiens | SH3GLB1 | up-regulated |
| + | 2.614951661 | Q9Y3Z3 | SAMH1_HUMAN | DeoxynucleosidetriphosphatetriphosphohydrolaseSAMHD1 | Homo sapiens | SAMHD1 | down-regulated |
| + | 2.108161482 | Q9Y570 | PPME1_HUMAN | Proteinphosphatasemethylesterase1 | Homo sapiens | PPME1 | up-regulated |
